# Mindfulness for smoking cessation

**DOI:** 10.1002/14651858.CD013696.pub2

**Published:** 2022-04-14

**Authors:** Sarah Jackson, Jamie Brown, Emma Norris, Jonathan Livingstone-Banks, Emily Hayes, Nicola Lindson

**Affiliations:** Department of Behavioural Science and HealthUniversity College LondonLondonUK; Health Behaviour Change Research GroupBrunel University LondonLondonUK; Nuffield Department of Primary Care Health SciencesUniversity of OxfordOxfordUK; Centre for Behaviour ChangeUniversity College LondonLondonUK

## Abstract

**Background:**

Mindfulness‐based smoking cessation interventions may aid smoking cessation by teaching individuals to pay attention to, and work mindfully with, negative affective states, cravings, and other symptoms of nicotine withdrawal. Types of mindfulness‐based interventions include mindfulness training, which involves training in meditation; acceptance and commitment therapy (ACT); distress tolerance training; and yoga.

**Objectives:**

To assess the efficacy of mindfulness‐based interventions for smoking cessation among people who smoke, and whether these interventions have an effect on mental health outcomes.

**Search methods:**

We searched the Cochrane Tobacco Addiction Group's specialised register, CENTRAL, MEDLINE, Embase, PsycINFO, and trial registries to 15 April 2021. We also employed an automated search strategy, developed as part of the Human Behaviour Change Project, using Microsoft Academic.

**Selection criteria:**

We included randomised controlled trials (RCTs) and cluster‐RCTs that compared a mindfulness‐based intervention for smoking cessation with another smoking cessation programme or no treatment, and assessed smoking cessation at six months or longer. We excluded studies that solely recruited pregnant women.

**Data collection and analysis:**

We followed standard Cochrane methods. We measured smoking cessation at the longest time point, using the most rigorous definition available, on an intention‐to‐treat basis. We calculated risk ratios (RRs) and 95% confidence intervals (CIs) for smoking cessation for each study, where possible. We grouped eligible studies according to the type of intervention and type of comparator. We carried out meta‐analyses where appropriate, using Mantel‐Haenszel random‐effects models. We summarised mental health outcomes narratively.

**Main results:**

We included 21 studies, with 8186 participants. Most recruited adults from the community, and the majority (15 studies) were conducted in the USA. We judged four of the studies to be at low risk of bias, nine at unclear risk, and eight at high risk. Mindfulness‐based interventions varied considerably in design and content, as did comparators, therefore, we pooled small groups of relatively comparable studies.

We did not detect a clear benefit or harm of mindfulness training interventions on quit rates compared with intensity‐matched smoking cessation treatment (RR 0.99, 95% CI 0.67 to 1.46; I^2^ = 0%; 3 studies, 542 participants; low‐certainty evidence), less intensive smoking cessation treatment (RR 1.19, 95% CI 0.65 to 2.19; I^2^ = 60%; 5 studies, 813 participants; very low‐certainty evidence), or no treatment (RR 0.81, 95% CI 0.43 to 1.53; 1 study, 325 participants; low‐certainty evidence). In each comparison, the 95% CI encompassed benefit (i.e. higher quit rates), harm (i.e. lower quit rates) and no difference. In one study of mindfulness‐based relapse prevention, we did not detect a clear benefit or harm of the intervention over no treatment (RR 1.43, 95% CI 0.56 to 3.67; 86 participants; very low‐certainty evidence).

We did not detect a clear benefit or harm of ACT on quit rates compared with less intensive behavioural treatments, including nicotine replacement therapy alone (RR 1.27, 95% CI 0.53 to 3.02; 1 study, 102 participants; low‐certainty evidence), brief advice (RR 1.27, 95% CI 0.59 to 2.75; 1 study, 144 participants; very low‐certainty evidence), or less intensive ACT (RR 1.00, 95% CI 0.50 to 2.01; 1 study, 100 participants; low‐certainty evidence). There was a high level of heterogeneity (I^2^ = 82%) across studies comparing ACT with intensity‐matched smoking cessation treatments, meaning it was not appropriate to report a pooled result.

We did not detect a clear benefit or harm of distress tolerance training on quit rates compared with intensity‐matched smoking cessation treatment (RR 0.87, 95% CI 0.26 to 2.98; 1 study, 69 participants; low‐certainty evidence) or less intensive smoking cessation treatment (RR 1.63, 95% CI 0.33 to 8.08; 1 study, 49 participants; low‐certainty evidence).

We did not detect a clear benefit or harm of yoga on quit rates compared with intensity‐matched smoking cessation treatment (RR 1.44, 95% CI 0.40 to 5.16; 1 study, 55 participants; very low‐certainty evidence).

Excluding studies at high risk of bias did not substantially alter the results, nor did using complete case data as opposed to using data from all participants randomised.

Nine studies reported on changes in mental health and well‐being, including depression, anxiety, perceived stress, and negative and positive affect. Variation in measures and methodological differences between studies meant we could not meta‐analyse these data. One study found a greater reduction in perceived stress in participants who received a face‐to‐face mindfulness training programme versus an intensity‐matched programme. However, the remaining eight studies found no clinically meaningful differences in mental health and well‐being between participants who received mindfulness‐based treatments and participants who received another treatment or no treatment (very low‐certainty evidence).

**Authors' conclusions:**

We did not detect a clear benefit of mindfulness‐based smoking cessation interventions for increasing smoking quit rates or changing mental health and well‐being. This was the case when compared with intensity‐matched smoking cessation treatment, less intensive smoking cessation treatment, or no treatment. However, the evidence was of low and very low certainty due to risk of bias, inconsistency, and imprecision, meaning future evidence may very likely change our interpretation of the results. Further RCTs of mindfulness‐based interventions for smoking cessation compared with active comparators are needed. There is also a need for more consistent reporting of mental health and well‐being outcomes in studies of mindfulness‐based interventions for smoking cessation.

## Plain language summary

**Can mindfulness help people to stop smoking?**

**Key messages**

‐ There is currently no clear evidence that mindfulness‐based treatments help people to stop smoking or improve their mental health and well‐being.

‐ However, our confidence in the evidence is low or very low, and further evidence is likely to change our conclusions.

**What is mindfulness?**

Mindfulness involves focusing attention on your thoughts and feelings and observing them without judgment as they arise and pass away. Mindfulness is believed to help people better control their thoughts and feelings, rather than be controlled by them. Stopping smoking gives rise to distressing urges to smoke and low mood, so mindfulness‐based treatments could improve people's ability to cope with these.

Types of mindfulness‐based therapies include:

‐ mindfulness training (which involves training in mindfulness‐based meditation);

‐ acceptance and commitment therapy (ACT); which doesn't teach meditation but encourages people to embrace their thoughts and feelings rather than fighting them, while making committed behaviour change);

‐ distress tolerance training (which provides parts of the ACT therapy, as well as presenting people who smoke with situations that make them want to smoke. This allows them to practise the skills that they have learnt through ACT);

‐ yoga (which increases awareness of breathing and encourages a connection between mind and body).

**What did we want to find out?**

We wanted to find out whether mindfulness‐based stop‐smoking programmes work better than other stop‐smoking programmes or no treatment to help people stop smoking.

We wanted to know:

‐ how many people stopped smoking for at least six months;

‐ whether there were changes in people's mental health and well‐being.

**What did we do?**

We searched for studies that looked at the use of mindfulness to help people stop smoking.

We compared and summarised the results of the studies and rated our confidence in the evidence, based on factors such as study methods and sizes.

**What did we find?**

We found 21 studies in 8186 young people and adults who smoked.

The studies tested a range of mindfulness‐based treatments, including mindfulness training (8 studies), ACT (8 studies), yoga (3 studies), and distress tolerance training (2 studies). Studies compared these treatments with:

‐ other stop‐smoking treatments that were equally time‐intensive (such as counselling);

‐ other stop‐smoking treatments that were less intensive (such as brief advice);

‐ no treatment.

Most studies took place in the USA (15 studies). Others took place in Hong Kong (2 studies), Brazil (1 study), Ireland (1 study), and Cyprus (1 study). One study did not report the country it took place in.

**Main results**

We did not find clear evidence that mindfulness helped people to stop smoking. When we grouped studies by the type of mindfulness‐based intervention people received, we found no evidence that people who received mindfulness training, ACT, distress tolerance training, or yoga were more likely to stop than people who received any other stop‐smoking treatments or no support.

Nine studies looked at whether mindfulness‐based stop‐smoking treatments resulted in positive changes in mental health and well‐being, such as reductions in stress or anxiety or improvements in mood. One of these studies found that people who received a mindfulness training programme reported being less stressed than those who received an alternative stop‐smoking treatment. However, the other 8 did not find evidence of a difference in mental health and well‐being between groups.

**What are the limitations of the evidence?**

Our confidence in the evidence is low to very low as there were problems with the design of studies, findings of studies were very different from one another, and not enough people took part, making it difficult to tell whether mindfulness helps people to stop smoking or was linked to better mental health and well‐being. We need more studies to draw firmer conclusions.

**How up to date is this evidence?**

We included evidence published to 15 April 2021.

## Summary of findings

**Summary of findings 1:** Mindfulness training compared with control for smoking cessation

**Mindfulness training compared with control for smoking cessation**						
**Patient or population:** people who smoke **Setting:** community; online; tobacco treatment services; high schools; workplaces (USA; Brazil; Hong Kong) **Intervention:** mindfulness training; mindfulness‐based relapse prevention **Comparisons:** matched‐intensity smoking cessation treatment; less intensive smoking cessation treatment; no treatment						
**Outcomes**	Anticipated absolute effects^*^ (95% CI)		**Relative effect (95% CI)**	**№ of participants (studies)**	**Certainty of the evidence (GRADE)**	**Comments**
	**Risk with control**	**Risk with mindfulness training**				
Mindfulness training vs matched‐intensity smoking cessation treatment: smoking cessation (≥ 6‐month follow‐up)	Study population		RR 0.99 (0.67 to 1.46)	542 (3 RCTs)	⊕⊕⊝⊝ Low^a,b^	
	16 per 100	16 per 100 (11 to 24)				
Mindfulness training vs less intensive smoking cessation treatment: smoking cessation (≥ 6‐month follow‐up)	Study population		RR 1.19 (0.65 to 2.19)	813 (5 RCTs)	⊕⊝⊝⊝ Very low^c,d,e^	
	11 per 100	14 per 100 (7 to 25)				
Mindfulness training vs no treatment: smoking cessation (≥ 6‐month follow‐up)	Study population		RR 0.81 (0.43 to 1.53)	325 (1 RCT)	⊕⊕⊝⊝ Low^f^	
	12 per 100	10 per 100 (5 to 18)				
Mindfulness‐based relapse prevention vs no treatment: smoking cessation (≥ 6‐month follow‐up)	Study population		RR 1.43 (0.56 to 3.67)	86 (1 RCT)	⊕⊝⊝⊝ Very low^g,h^	
	14 per 100	20 per 100 (8 to 52)				
Mental health and well‐being	Studies investigated a range of outcomes: anxiety, depression, negative affect, positive affect, stress. Although 1 study found a statistically significantly greater reduction in perceived stress in people who received mindfulness training compared with those who received a matched‐intensity smoking cessation treatment at 6‐month follow‐up, the other 2 studies found no clinically meaningful between‐group differences in change in mental health and well‐being measures.			633 (3 RCTs)	⊕⊝⊝⊝ Very low^i,j^	We were unable to meta‐analyse these outcomes and therefore summarised them narratively.
*The risk in the intervention group (and its 95% confidence interval) is based on the assumed risk in the comparison group and the **relative effect** of the intervention (and its 95% CI). **CI:** confidence interval; **RCT:** randomised controlled trial; **RR:** risk ratio						
**GRADE Working Group grades of evidence** **High certainty:** we are very confident that the true effect lies close to that of the estimate of the effect. **Moderate certainty:** we are moderately confident in the effect estimate: the true effect is likely to be close to the estimate of the effect, but there is a possibility that it is substantially different. **Low certainty:** our confidence in the effect estimate is limited: the true effect may be substantially different from the estimate of the effect. **Very low certainty:** we have very little confidence in the effect estimate: the true effect is likely to be substantially different from the estimate of effect.						

^a^Not downgraded for risk of bias: we judged one of the three studies to be at high risk of bias, but excluding this study did not change the conclusion. ^b^Downgraded by two levels due to imprecision: the overall number of events was very low (n = 87) and the confidence interval of the effect estimate incorporates clinically relevant potential benefit and harm of the intervention. ^c^Downgraded by one level due to risk of bias: we judged two of the five studies to be at high risk of bias and removing these studies changed the direction of the effect estimate. ^d^Downgraded by one level due to inconsistency: substantial unexplained heterogeneity (I² = 60%). ^e^Downgraded by two levels due to imprecision: the overall number of events was low (n = 101) and the confidence interval of the effect estimate incorporates clinically relevant potential benefit and harm of the intervention. ^f^Downgraded by two levels due to imprecision: the overall number of events was very low (n = 36) and the confidence interval of the effect estimate incorporates clinically relevant potential benefit and harm of the intervention. ^g^Downgraded by two levels due to risk of bias: we judged the sole study to be at high risk of bias. ^h^Downgraded by two levels due to imprecision: the overall number of events was very low (n = 15) and the confidence interval of the effect estimate incorporates clinically relevant potential benefit and harm of the intervention. ^i^Downgraded by one level due to risk of bias: we judged two of the three studies to be at high risk of bias and one of these was the only study to report a meaningful difference in mental health between conditions. ^j^Downgraded by two levels due to inconsistency: mental health and well‐being are measured using a range of different constructs, the interventions include both a standard cessation intervention and an intervention targeted at relapse prevention and the interpretation of results varies across studies.

**Summary of findings 2:** Acceptance and commitment therapy (ACT) compared with control for smoking cessation

**Acceptance and commitment therapy (ACT) compared with control for smoking cessation**						
**Patient or population:** people who smoke **Setting:** community; online; primary care; high schools and universities (USA; Cyprus; Hong Kong; Ireland) **Intervention:** Acceptance and commitment therapy (ACT) **Comparisons:** matched‐intensity smoking cessation treatment; NRT; brief advice; less intensive ACT						
**Outcomes**	Anticipated absolute effects^*^ (95% CI)		**Relative effect (95% CI)**	**№ of participants (studies)**	**Certainty of the evidence (GRADE)**	**Comments**
	**Risk with control**	**Risk with ACT**				
ACT vs matched‐intensity smoking cessation treatment: smoking cessation (≥ 6‐month follow‐up)	It was not appropriate to pool data across these studies because there was a high level of heterogeneity (I^2^ = 82%) and the result may be misleading.			5723 (5 RCTs)	⊕⊝⊝⊝ Very low^a,b,c^	
ACT vs NRT: smoking cessation (≥ 6‐month follow‐up)	Study population		RR 1.27 (0.53 to 3.02)	102 (1 RCT)	⊕⊕⊝⊝ Low^d^	
	15 per 100	19 per 100 (8 to 45)				
ACT vs brief advice: smoking cessation (≥ 6‐month follow‐up)	Study population		RR 1.27 (0.59 to 2.75)	144 (1 RCT)	⊕⊝⊝⊝ Very low^d,e^	
	14 per 100	17 per 100 (8 to 37)				
ACT vs less intensive ACT: smoking cessation (≥ 6‐month follow‐up)	Study population		RR 1.00 (0.50 to 2.01)	100 (1 RCT)	⊕⊕⊝⊝ Low^d^	
	24 per 100	24 per 100 (12 to 48)				
Mental health and well‐being	One study that compared ACT with NRT found no clinically meaningful difference in negative affect across conditions at all follow‐ups to 12 months. Another study that compared ACT with a matched‐intensity smoking cessation treatment and a less intensive ACT intervention found no clinically meaningful difference in positive mental health across conditions up to 6‐month follow‐up.			252 (2 RCTs)	⊕⊝⊝⊝ Very low^f,g^	We were unable to meta‐analyse this outcome and therefore summarised narratively.
*The risk in the intervention group (and its 95% confidence interval) is based on the assumed risk in the comparison group and the **relative effect** of the intervention (and its 95% CI). **ACT:** Acceptance and commitment therapy; **CI:** confidence interval; **NRT:** nicotine replacement therapy; **RCT:** randomised controlled trial; **RR:** risk ratio						
**GRADE Working Group grades of evidence** **High certainty:** we are very confident that the true effect lies close to that of the estimate of the effect. **Moderate certainty:** we are moderately confident in the effect estimate: the true effect is likely to be close to the estimate of the effect, but there is a possibility that it is substantially different. **Low certainty:** our confidence in the effect estimate is limited: the true effect may be substantially different from the estimate of the effect. **Very low certainty:** we have very little confidence in the effect estimate: the true effect is likely to be substantially different from the estimate of effect.						

^a^Downgraded by two levels due to inconsistency: substantial heterogeneity was detected (I² = 82%). A subgroup analysis grouping by mode of delivery used explained a small amount of this, but substantial heterogeneity remained unexplained. ^b^Not downgraded for indirectness. One study included only smokers without health insurance, but contributed just 17.3% of the weighted effect. ^c^Downgraded by one level due to imprecision: the confidence interval of the effect estimate incorporates clinically relevant potential benefit and harm of the intervention. ^d^Downgraded by two levels due to imprecision: the overall number of events was very low (< 25) and the confidence interval of the effect estimate incorporates clinically relevant potential benefit and harm of the intervention. ^e^Downgraded by two levels due to risk of bias: we judged the sole study to be at high risk of bias. ^f^Downgraded by two levels due to inconsistency: the constructs and measures of mental health used differed across studies, as well as the study comparators. ^g^Downgraded by two levels due to imprecision: two small studies likely lacked sufficient statistical power to detect clinically meaningful effects.

**Summary of findings 3:** Distress tolerance training compared with control for smoking cessation

**Distress tolerance training compared with control for smoking cessation**						
**Patient or population:** people who smoke **Setting:** community (USA) **Intervention:** distress tolerance training **Comparisons:** matched‐intensity smoking cessation treatment; less intensive smoking cessation treatment						
**Outcomes**	Anticipated absolute effects^*^ (95% CI)		**Relative effect (95% CI)**	**№ of participants (studies)**	**Certainty of the evidence (GRADE)**	**Comments**
	**Risk with control**	**Risk with distress tolerance training**				
Distress tolerance training vs matched‐intensity smoking cessation treatment: smoking cessation (≥ 6‐month follow‐up)	Study population		RR 0.87 (0.26 to 2.98)	69 (1 RCT)	⊕⊕⊝⊝ Low^a^	
	14 per 100	12 per 100 (4 to 41)				
Distress tolerance training vs less intensive smoking cessation treatment: smoking cessation (≥ 6‐month follow‐up)	Study population		RR 1.63 (0.33 to 8.08)	49 (1 RCT)	⊕⊕⊝⊝ Low^a^	
	9 per 100	15 per 100 (3 to 73)				
Mental health and well‐being	One study that compared distress tolerance training with less intensive smoking cessation treatment found no clinically meaningful difference in negative affect at 4 weeks post‐quit.			49 (1 RCT)	⊕⊕⊝⊝ Low^b^	We were unable to meta‐analyse this outcome and therefore summarised narratively.
*The risk in the intervention group (and its 95% confidence interval) is based on the assumed risk in the comparison group and the **relative effect** of the intervention (and its 95% CI). **CI:** confidence interval; **RCT:** randomised controlled trial; **RR:** risk ratio						
**GRADE Working Group grades of evidence** **High certainty:** we are very confident that the true effect lies close to that of the estimate of the effect. **Moderate certainty:** we are moderately confident in the effect estimate: the true effect is likely to be close to the estimate of the effect, but there is a possibility that it is substantially different. **Low certainty:** our confidence in the effect estimate is limited: the true effect may be substantially different from the estimate of the effect. **Very low certainty:** we have very little confidence in the effect estimate: the true effect is likely to be substantially different from the estimate of effect.						

^a^Downgraded by two levels due to imprecision: the overall number of events was very low (< 10) and the confidence interval of the effect estimate incorporates clinically relevant potential benefit and harm of the intervention. ^b^Downgraded by two levels due to imprecision: the overall number of participants was very low (< 50).

**Summary of findings 4:** Yoga compared with control for smoking cessation

**Yoga compared with control for smoking cessation**						
**Patient or population:** people who smoke **Setting:** community (USA) **Intervention:** yoga **Comparison:** matched‐intensity smoking cessation treatment; less intensive smoking cessation treatment						
**Outcomes**	Anticipated absolute effects^*^ (95% CI)		**Relative effect (95% CI)**	**№ of participants (studies)**	**Certainty of the evidence (GRADE)**	**Comments**
	**Risk with control**	**Risk with yoga**				
Yoga vs matched‐intensity smoking cessation treatment: smoking cessation (≥ 6‐month follow‐up)	Study population		RR 1.44 (0.40 to 5.16)	55 (1 RCT)	⊕⊝⊝⊝ Very low^a,b^	
	13 per 100	19 per 100 (5 to 67)				
Mental health and well‐being	One study compared yoga with matched‐intensity smoking cessation treatment and found no clinically meaningful difference in depression, anxiety, or general well‐being scores between conditions at 8‐week follow‐up after controlling for baseline scores. Another study compared yoga with less intensive smoking cessation treatment and found no clinically meaningful differences in the change in depression or anxiety scores by group up to 6‐month follow‐up.			93 (2 RCTs)	⊕⊝⊝⊝ Very low^c,d^	We were unable to meta‐analyse this outcome and therefore summarised narratively.
*The risk in the intervention group (and its 95% confidence interval) is based on the assumed risk in the comparison group and the **relative effect** of the intervention (and its 95% CI). **CI:** confidence interval; **RCT:** randomised controlled trial; **RR:** risk ratio						
**GRADE Working Group grades of evidence** **High certainty:** we are very confident that the true effect lies close to that of the estimate of the effect. **Moderate certainty:** we are moderately confident in the effect estimate: the true effect is likely to be close to the estimate of the effect, but there is a possibility that it is substantially different. **Low certainty:** our confidence in the effect estimate is limited: the true effect may be substantially different from the estimate of the effect. **Very low certainty:** we have very little confidence in the effect estimate: the true effect is likely to be substantially different from the estimate of effect.						

^a^Downgraded by two levels due to risk of bias: we judged the sole study to be at high risk of bias. ^b^Downgraded by two levels due to imprecision: the overall number of events was very low (< 10) and the confidence interval of the effect estimate incorporates clinically relevant potential benefit and harm of the intervention. ^c^Downgraded by two levels due to risk of bias: we judged both studies to be at high risk of bias. ^d^Downgraded by two levels due to imprecision: two small studies likely lacked sufficient statistical power to detect clinically meaningful effects

## Background

### Description of the condition

Smoking remains a leading cause of preventable death and disease worldwide (WHO 2019). Stopping smoking can result in substantial health gains, even later in life. The sooner a smoker quits, the more they reduce their risk of developing smoking‐related diseases (Doll 2004). The majority of smokers want to quit and many try to quit each year, but quit rates remain low (WHO 2019).

### Description of the intervention

In recent decades, mindfulness has increasingly been recognised as an influence on mood and behaviour (Baer 2003; Keng 2011). It has been adopted as an approach for increasing awareness and responding skilfully to mental processes that contribute to emotional distress and maladaptive behaviour (Baer 2003). In current research contexts, mindfulness is typically defined as the psychological process of bringing non‐judgmental attention to experiences occurring in the present moment (Kabat‐Zinn 2013). There are various definitions of mindfulness used in psychological literature. While no consensus has been reached on how to define mindfulness, a two‐component model proposed by Bishop 2004 is often used in research. This operationalises mindfulness as: (i) maintaining attention on the immediate experience, and (ii) maintaining an attitude of openness, curiosity, and acceptance toward this experience, regardless of its valence or desirability.

Mindfulness approaches are not relaxation or mood management techniques, but rather a form of cognitive training to reduce susceptibility to reactive states of mind that might otherwise induce stress or perpetuate psychopathology (Baer 2003). The practice of mindfulness involves focusing attention on the immediate experience of cognitions, emotions, perceptions, and physical sensations and observing them as they arise and pass away. Mindfulness is nondeliberative: it simply involves paying sustained attention to thoughts and feelings without thinking about or evaluating them. A key tenet of mindfulness is that, by noticing thoughts and feelings in a curious and accepting manner, people develop greater tolerance of these phenomena and are able to recognise that they are transient, so they are less likely to respond impulsively to them (Heppner 2015).

There are a range of different treatments based on the principles of mindfulness. Mindfulness‐based stress reduction (MBSR; Kabat‐Zinn 2013) and mindfulness‐based cognitive therapy (MBCT; Segal 2002) use meditation as the primary method of teaching mindfulness. MBSR was developed to treat chronic stress and pain‐related disorders. It uses three techniques: firstly, sitting meditation, which involves mindful attention on the breath and a state of noncritical awareness of cognitions, feelings, and sensations; secondly, Hatha yoga practice, which involves breathing exercises, simple stretches, and postures; and thirdly, body scan, which involves a gradual sweeping of attention through the entire body from feet to head, while employing nonjudgmental awareness of feelings and sensation in each targeted body region (Kabat‐Zinn 2013). MBCT was developed to prevent relapse in depressive disorders. It integrates aspects of cognitive behavioral therapy (CBT) for depression into the MBSR programme (Segal 2002).

Other treatments that incorporate mindfulness include acceptance and commitment therapy (ACT; Hayes 2016), distress tolerance training, dialectical behaviour therapy (DBT; Linehan 2018), and certain types of yoga (Salmon 2009). ACT focuses on increasing people's willingness to experience physical cravings, emotions, and thoughts, and allowing these to come and go while making committed behaviour changes that are guided by their own values (Hayes 2016). Distress tolerance training combines elements drawn from ACT with exposure‐based treatment, allowing ACT skills to be practised within treatment sessions in response to internal triggers (Brown 2008). DBT also has a strong emphasis on acceptance, incorporating strategies to help the patient accept themselves, their current capabilities, and behavioural functioning (Linehan 2018). Yoga is a key component of MBSR (Kabat‐Zinn 2013), and provides an opportunity to practise mindfulness through movement. Forms of yoga that incorporate breathing exercises and directed meditative focus work to still the mind and focus attention (Bock 2012).

### How the intervention might work

Mindfulness‐based interventions may aid smoking cessation by teaching individuals to pay attention to, and work mindfully with, negative affective states, cravings, and other symptoms of nicotine withdrawal as they arise, rather than habitually reacting to these unpleasant states by smoking. Proposed mechanisms of action include attention regulation, body awareness, emotion regulation, and change in self‐perspective (Hölzel 2011).

Withdrawal following smoking cessation is acutely associated with heightened levels of stress and negative affect (Shiffman 2004; West 2017). Once withdrawal symptoms have abated, cessation is generally associated with improved mental health (Taylor 2014; Taylor 2021), but early stage acute stress, negative affect, and depression are predictive of relapse (Correa‐Fernández 2012; Glassman 1990; Shiffman 2004; Shiffman 2005). Therefore, interventions that work to reduce these adverse emotional consequences of stopping smoking may enhance quit rates and ultimately prevent relapse. Mindfulness‐based interventions have shown some efficacy in the treatment of psychiatric disorders relating to or involving these negative affective states (Goyal 2014; Marchand 2013).

Further, by teaching smokers to focus their attention on what is happening in the moment, mindfulness‐based interventions bring habitual behaviours into consciousness. This enables people to understand the associative learning process, and focus on affect and craving as central components of positive and negative reinforcement loops (Brewer 2010). By emphasising the transience of affective states and teaching smokers to ‘sit with’ negative affect and craving, mindfulness interventions target and modify learned responses to smoking cues. This may help smokers to quit and may reduce cigarette consumption among those who do not stop smoking completely.

Thus, it has been suggested that mindfulness‐based treatments “may have the relative advantage of teaching a single technique that may lead to the dampening and eventual dismantling of the complex interrelated associative processes of smoking rather than just removing stimuli that might propagate them” (Brewer 2011).

### Why it is important to do this review

If found to be effective, mindfulness‐based interventions could add an innovative intervention option to the range of treatments for smoking cessation. A systematic review, including literature to 2016, did not find evidence of a significant impact of mindfulness meditation interventions on abstinence relative to comparator groups (Maglione 2017). However, the evidence identified was of low certainty due to the high levels of heterogeneity and imprecision detected through meta‐analysis. Therefore, there is a need to update this review to include new evidence, in an effort to increase the certainty of the resulting conclusions. In addition, expanding the search to include other interventions that incorporate mindfulness approaches but do not specifically include an element of meditation (e.g. ACT) can add to our understanding of the potential effectiveness of mindfulness for smoking cessation.

The purpose of the present review is to assess the effect of interventions that incorporate mindfulness approaches for smoking cessation, using the robust methodology of Cochrane and the Cochrane Tobacco Addiction Group. This review also represents part of a separate project to evaluate similarities and differences between the standard methodological processes of the Cochrane Tobacco Addiction Group and a novel, machine‐learning approach developed by the Human Behaviour Change Project (Michie 2013).

## Objectives

To assess the efficacy of mindfulness‐based interventions for smoking cessation among people who smoke, and whether these interventions have an effect on mental health outcomes.

## Methods

### Criteria for considering studies for this review

#### Types of studies

Randomised controlled trials (RCTs) and cluster‐RCTs that measured smoking cessation at least six months from baseline were eligible for this review. We included studies reported as full text, those published as abstract only, and unpublished data, where available. There were no language or date restrictions.

#### Types of participants

We included current tobacco smokers of any age who were willing to enrol in a smoking cessation study. We excluded studies that only recruited pregnant women, as their particular needs and circumstances warrant their treatment as a separate population, and these are covered in a separate Cochrane Review (Chamberlain 2017).

#### Types of interventions

We included interventions targeted at tobacco smoking cessation that were either labelled as mindfulness, or involved a mindfulness‐based approach that could be isolated to investigate effectiveness. There were no restrictions on the minimum duration of the intervention. Where a potentially relevant study intervention was not specifically described as being mindfulness‐based, we discussed as a team (of EN, JLB, NL, SJ) whether it was eligible for inclusion. We intentionally adopted an inclusive approach, including interventions that incorporated mindfulness (e.g. ACT or yoga) in addition to those specifically focused on mindfulness meditation (e.g. MBSR or MBCT) to capture the broadest evidence.

Eligible studies had to include at least one of the following comparison (control) interventions:

- no smoking cessation treatment;
- another smoking cessation intervention, of any length or intensity (including usual care);
- another type of mindfulness intervention (e.g. mindfulness of a lower intensity).

#### Types of outcome measures

##### Primary outcomes

###### Smoking abstinence at longest follow‐up

To be eligible for inclusion, studies must have measured abstinence at least six months from the start of the intervention. Following the Cochrane Tobacco Addiction Group's standard methods, we excluded studies that only measured abstinence at less than six‐months' follow‐up.

In studies with more than one measure of abstinence, we preferred the measure with the strictest criteria, in line with the Russell Standard (West 2005). We used prolonged or continuous abstinence in preference to point prevalence abstinence, and preferred biochemically validated abstinence (e.g. using exhaled carbon monoxide or cotinine measures) over self‐report. We favoured biochemically validated point prevalence abstinence over self‐reported continuous or prolonged abstinence.

###### Mental health and well‐being

This could provide us with information on potential benefits or harms of the mindfulness‐based interventions. Even if comparisons of mindfulness‐based interventions with other smoking cessation interventions do not find a benefit of mindfulness for smoking cessation, improved mental well‐being could be a reason for choosing this treatment over another. We assessed validated measures of the following relevant constructs:

- depression;
- anxiety;
- quality of life;
- positive affect;
- negative affect;
- stress.

We extracted data on these mental health and well‐being outcomes, measured at the longest follow‐up at which abstinence was reported, or as close to this as possible.

### Search methods for identification of studies

#### Electronic searches

We searched the following databases for studies that referred to mindfulness techniques in the title or abstract, or as keywords:

- Cochrane Tobacco Addiction Group Specialised Register via the Cochrane Register of Studies (CRS‐Web)
- Cochrane Central Register of Controlled Trials (CENTRAL; 2021, issue 3) via CRS‐Web
- MEDLINE Ovid (1946 to 15 April 2021)
- Embase Ovid (1974 to 15 April 2021)
- PsycINFO Ovid (1806 to 15 April 2021)

We searched all databases from inception through to 15 April 2021. At the time of the search, the Register included the results of searches of MEDLINE (via OVID) to update 20210407; Embase (via OVID) to week 202114; PsycINFO (via OVID) to update 20210329. See the Tobacco Addiction Group website for details of the search strategies for these databases. Search strategies are shown in Appendix 1. 

By searching CENTRAL and the Cochrane Tobacco Addiction Group’s register, we were able to identify any ongoing studies registered in the World Health Organization's portal (www.who.int/trialsearch) or ClinicalTrials.gov in the USA, and studies reported in Annual Meeting abstracts for the Society for Research on Nicotine and Tobacco (SRNT). We listed in the Characteristics of ongoing studies table any studies that may be candidates for inclusion (i.e. RCTs of smoking cessation interventions using mindfulness‐based approaches with a minimum follow‐up of six months), but for which results are not yet available.

#### Searching other resources

We checked reference lists of eligible published papers to identify any other relevant papers that may not have been identified by our search, and consulted experts in the field to identify any relevant forthcoming or unpublished research. We contacted the authors of ongoing studies where necessary.

Alongside these manual search strategies, we employed an automated search strategy developed as part of the Human Behaviour Change Project (Michie 2017), using Microsoft Academic. The Human Behaviour Change Project aims to improve upon the human ability to synthesise, interpret and deliver evidence on behaviour change interventions, using Natural Language Processing and Machine Learning technologies to automate the extraction, synthesis, and interpretation of findings from behaviour change intervention evaluation reports. We added any additional studies identified through this method to those found via the manual search, so that we included all relevant evidence. An evaluation comparing these manual and automated methods of study identification will be reported in a separate paper.

### Data collection and analysis

#### Selection of studies

Two review authors (of EN, JLB, NL, SJ), independently checked the titles and abstracts of retrieved studies for relevance, and acquired full study reports of those that may be candidates for inclusion. The review authors resolved any disagreements by mutual consent, or by recourse to a third review author. Two review authors (of EN, JLB, NL, SJ) then independently assessed the full texts for eligibility, resolving any disagreements through discussion and with involvement of a third review author when necessary. We classified as 'exclude' any studies for which we obtained full reports, but that did not meet the inclusion criteria.

#### Data extraction and management

Two review authors (of EH, EN, JLB, NL, SJ) independently extracted study data and compared their findings. We resolved any disagreements through discussion, involving a third review author where necessary. Where available, we recorded the following information in the Characteristics of included studies table.

- Methods: study design, study name (if applicable), study dates, country, number of study centres, study setting, study recruitment procedure
- Participants: number (intervention/control), definition of smoker used, specific demographic characteristics (e.g. mean age, age range, gender, ethnicity, socioeconomic status (SES)), mean cigarettes per day, mean Fagerstrom Test for Nicotine Dependence (FTND), relevant inclusion and exclusion criteria
- Interventions: description of intervention(s) (details of behavioural support and any pharmacological treatment provided), description of control (details of behavioural support and any pharmacological treatment provided), what comparisons will be constructed between which groups
- Outcomes: relevant primary and secondary outcomes measured, time points reported, biochemical validation, definitions of abstinence, mental health measures used, proportion of participants with follow‐up data
- Details of any within‐study analyses of moderators of interest: population type; baseline motivation to quit; baseline mental health
- Notes: funding for study, and conflicts of interest statements of study authors (extracted verbatim)

Alongside this data extraction of entities that are typically captured in smoking cessation Cochrane Reviews, we also performed data extraction using entities of the Behaviour Change Intervention Ontology, which is being developed as part of the Human Behaviour Change Project (Michie 2017). The ontology consists of granular entities to specify all aspects of behaviour change interventions, such as:

- an intervention’s context (including 'setting' (Norris 2020) and 'population');
- content (including 'behaviour change techniques'; (Michie 2013)); and
- delivery (including 'mode of delivery': how an intervention is provided to participants (Marques 2021); 'source': who delivers interventions (Norris 2021); and 'schedule': how often an intervention is delivered (Michie 2017)).

An evaluation to compare these methods of data extraction will be reported in a separate paper.

#### Assessment of risk of bias in included studies

Two review authors (of JLB, NL, SJ) independently assessed the risk of bias for each included study. We used RoB 1, following the guidance as set out in the *Cochrane Handbook for Systematic Reviews of Interventions* (Higgins 2011).

We assessed the following domains (Higgins 2011):

- sequence generation;
- allocation concealment,
- blinding of outcome assessment;
- incomplete outcome data;
- selective reporting; and
- other sources of bias.

As we were investigating a primarily behavioural intervention, we did not assess the blinding of participants and providers, as it is impossible to blind people to behavioural interventions. This is in accordance with specific guidance from the Cochrane Tobacco Addiction Group.

Each review author recorded information in study reports relevant to each domain and then assessed each domain as either at low, high, or unclear risk of bias. We resolved disagreements by discussion with a third review author. We considered studies to be at high overall risk of bias where we judged at least one domain to be at high risk; at low overall risk of bias where all domains were judged to be at low risk; and at unclear overall risk of bias in all other cases. 

#### Measures of treatment effect

We compared quit rates between intervention and comparator groups for each study. We calculated quit rates on an intention‐to‐treat basis, including all participants originally randomised to a study arm, treating participants lost to follow‐up as relapsed. We calculated a risk ratio (RR) and 95% confidence interval (CI) for each study. We calculated the RR for each study as: (number of participants who reported smoking abstinence in the intervention group/number of participants randomised to the intervention group)/(number of participants who reported smoking abstinence in the control (comparator) group/number of participants randomised to the control (comparator) group).

Due to high levels of variance between studies in interventions and comparators, and in the measurement of mental health and well‐being outcomes, we narratively reported relevant measures of mental health and well‐being.

#### Unit of analysis issues

The one included cluster‐RCT did not present an analysis adjusting for the clustering effect or report an intracluster correlation coefficient (ICC). Therefore, we used unadjusted data for the primary analysis and performed a sensitivity analysis where we estimated the ICC (0.03), based on the ICC reported in other smoking cessation studies (Fanshawe 2017), and adjusted the analysis on this basis.

In the case of studies with multiple intervention arms, we analysed individual arms separately.

#### Dealing with missing data

For smoking abstinence, we assumed participants lost to follow‐up to be smoking, as is standard in the field (West 2005). However, we conducted a sensitivity analysis, excluding numbers lost to follow‐up from the denominator. 

#### Assessment of heterogeneity

In order to assess whether it was appropriate to pool studies and conduct meta‐analyses, we assessed the characteristics of included studies to identify any clinical or methodological variance between studies. If we deemed the studies to be homogeneous enough to be combined meaningfully and we could conduct meta‐analyses, we assessed statistical heterogeneity using the I^2^ statistic (Higgins 2003). We considered an I^2^ statistic over 50% to indicate moderate to substantial heterogeneity (Deeks 2021). Where the I^2^ statistic was 80% or more, the direction of individual study effects differed, and heterogeneity was not fully explained by subgroup and sensitivity analyses, we do not report a pooled estimate because it could be misleading. We conducted the subgroup and sensitivity analyses described below to investigate any potential causes of observed heterogeneity.

#### Assessment of reporting biases

It was not appropriate to assess reporting bias using funnel plots as none of our analyses pooled 10 or more studies.

#### Data synthesis

We provided a narrative summary of the included studies and, where appropriate, conducted meta‐analyses.

The primary outcome of abstinence provides dichotomous data, therefore, as per the Cochrane Tobacco Addiction Group's standard methods, we combined RRs from individual studies using random‐effects, Mantel‐Haenszel methods, to calculate pooled overall RRs with 95% CIs.

Meaures of our mental health and well‐being outcome typically provided continuous data. Data were too heterogeneous to carry out meta‐analyses, so we tabulated the existing information and summarised narratively. 

We also narratively reported the results of any within‐study analyses that have investigated the following moderators of effectiveness at at least six months' follow‐up:

- population type;
- baseline motivation to quit;
- baseline mental health.

#### Subgroup analysis and investigation of heterogeneity

We carried out subgroup analyses, categorising studies by the type/intensity of control treatment received and mode of intervention delivery. We compared pooled summary statistics across groups and ran statistical tests for subgroup differences.

#### Sensitivity analysis

For smoking abstinence, we tested the impact of excluding studies deemed to be at overall high risk of bias and compared abstinence rates calculated assuming 'missing equals smoking' with abstinence rates calculated through complete‐case analysis. We also carried out the sensitivity analysis reported above, using an assumed ICC to adjust for potential clustering effects in a cluster‐RCT.

#### Summary of findings and assessment of the certainty of the evidence

Following standard Cochrane methodology (Schünemann 2021), we created summary of findings tables for smoking abstinence, and mental health and well‐being outcomes, detailing different intervention types in separate tables (mindfulness training; ACT; distress tolerance training; yoga). Also following standard Cochrane methodology (Schünemann 2021), we used the five GRADE considerations (risk of bias, inconsistency, imprecision, indirectness, and publication bias) to assess the certainty of the body of evidence for each outcome, within each comparison, and to draw conclusions about the certainty of evidence within the text of the review.

## Results

### Description of studies

#### Results of the search

Our bibliographic database searches and automated search process identified 2900 non‐duplicate records (Figure 1). We screened all records and retrieved the full‐text papers of 166 potentially relevant articles. After screening and checking the full texts, we identified 57 reports relating to 27 studies. Of these, 21 were completed studies (see Characteristics of included studies table) and six were ongoing studies (see Characteristics of ongoing studies table). We were unable to classify one study because the follow‐up period was unclear (see Characteristics of studies awaiting classification table).

**1:**
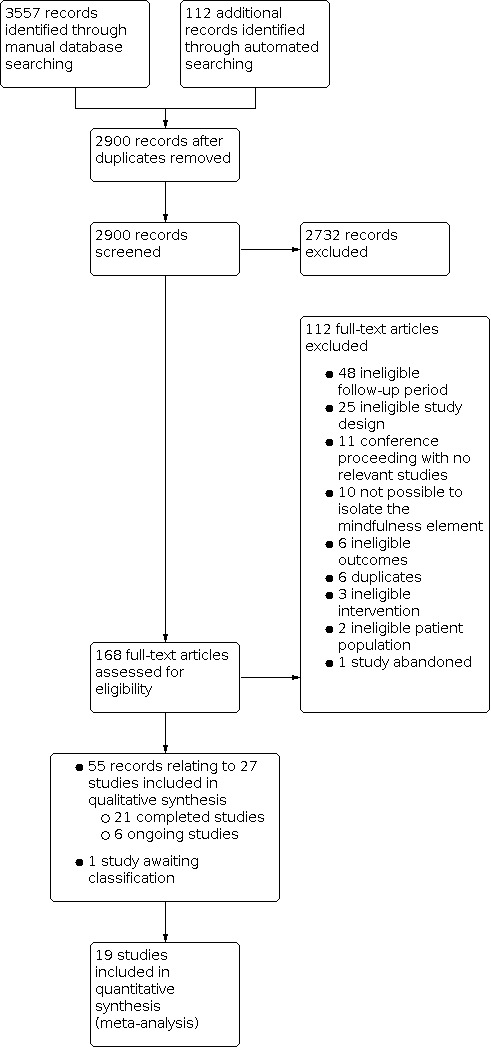
Figure 1: PRISMA flow diagram

#### Included studies

 In total, we included 21 completed studies (Bloom 2020; Bock 2012; Bock 2019; Bricker 2014a; Bricker 2018; Bricker 2020; Brown 2013; Davis 2014a; Davis 2014b; de Souza 2020; Garrison 2020; Gaskins 2015; Gifford 2003; Mak 2020; McClure 2020; O'Connor 2020; Pbert 2020; Savvides 2014; Singh 2014; Vidrine 2016; Weng 2021). Key features of the included studies are summarised below. 

##### Context and participants

Studies were conducted in the USA (15 studies), Hong Kong (2 studies), Brazil (1 study), Ireland (1 study), and Cyprus (1 study). One study (Singh 2014) did not report its location. Participants were recruited from the community (12 studies), online (3 studies), from healthcare centres (2 studies), high schools and universities (2 studies), tobacco treatment services (1 study), and workplaces (1 study). One study was a cluster‐RCT (Pbert 2020), which randomised high schools to different conditions. All other studies were randomised at the individual level.

The total number of participants across studies was 8186. The median sample size was 146 but ranged from 38 to 2637 participants. Two studies deliberately targeted young adults (Pbert 2020; Savvides 2014), two studies low SES smokers (Davis 2014a; Davis 2014b), one study uninsured smokers (Bricker 2014a), one study smokers with a history of early lapse (never able to remain abstinent for more than 72 hours; Brown 2013), and one study adults with mild intellectual disability (Singh 2014). Most studies had similar proportions of men and women or slightly more women than men. The exceptions were Bloom 2020, Bock 2012 and Weng 2021, which targeted only women; Gaskins 2015, which targeted only men; Singh 2014, which recruited 82% men with mild intellectual disability; and Mak 2020, which was conducted in Hong Kong where smoking prevalence among women is low and recruited 71% men. 

Studies typically recruited people who smoked at least five cigarettes a day. Although some studies included lighter smokers as well, the average number smoked was over 15 a day in most studies, ranging from five a day in Pbert 2020's sample of high school students to 22 a day in Brown 2013's community sample. 

##### Intervention programmes

###### Mindfulness training

Eight studies used mindfulness training (which, for the purpose of this review, we define as specific training in mindfulness and mindfulness‐based meditation techniques).

Five studies tested the effectiveness of mindfulness training delivered face‐to‐face. Davis 2014b compared seven weeks of group mindfulness training and meditation practice with an alternative, intensity‐matched, behavioural support programme. Similarly, Vidrine 2016 compared mindfulness‐based addiction treatment (8 x 2‐hour sessions) with an intensity‐matched CBT programme. In the latter study, there was also a second, less intensive comparator arm, in which participants received briefer support intended to represent the intervention a smoker might typically receive if they asked a healthcare provider for help (4 x 5‐ to 10‐minute sessions). All participants received self‐help materials. The other three studies compared mindfulness training with less intensive comparators. Davis 2014a compared an eight‐week mindfulness and meditation training programme with quitline support. Singh 2014 compared mindfulness and meditation training for adults with mild intellectual disability with treatment as usual, which varied between participants and encompassed a range of treatments such as behaviour therapies, nicotine replacement therapy (NRT), and other medications. Weng 2021 provided women in workplaces with self‐help materials and compared the effectiveness of additional mindfulness and meditation training (2 x 2‐hour sessions), with brief advice to follow the advice of the self‐help materials. Provision of pharmacotherapy varied between studies: three studies (Davis 2014a; Davis 2014b; Vidrine 2016), provided participants in both arms with a course of nicotine patches, one study provided no pharmacotherapy (Weng 2021), and one study (Singh 2014), did not specifically provide pharmacotherapy to participants in either arm, although for some comparator arm participants it was part of their usual treatment.

Two studies tested the effectiveness of mindfulness training delivered via smartphone apps. Garrison 2020 was conducted online. It compared a mobile mindfulness training app plus experience sampling (which asked participants to check in 6 times a day for 22 days) with experience sampling only. Pbert 2020 was a cluster‐RCT conducted in high schools. It tested the effectiveness of a mindfulness smartphone app designed for teens against two comparators: firstly, an alternative (non‐mindfulness) smoking cessation app designed for teens and secondly, self‐help materials. Participants in each of the three arms met with the school nurse weekly for four weeks. No pharmacotherapy was provided in either study.

de Souza 2020 was the only study to focus on mindfulness for relapse prevention. All participants received CBT over two phases: a smoking cessation phase (weekly sessions over 4 weeks) and a maintenance phase (6 sessions between weeks 6 and 48). The intervention arm also received eight mindfulness‐based relapse prevention sessions during the maintenance phase. Participants were offered the choice of NRT or bupropion.

Behaviour‐change techniques (BCTs) varied across studies. The most commonly used techniques included body changes (7 studies), problem solving (4 studies), self‐monitoring of behaviour (4 studies), pharmacological support (4 studies), and goal setting (3 studies), with no clear patterning in the number or type of BCTs used across mode of delivery.

###### Acceptance and commitment therapy (ACT)

Eight studies used ACT.

Three studies tested the effectiveness of ACT delivered exclusively face‐to‐face. McClure 2020 compared a five‐week, group ACT programme with a five‐week, group CBT programme. The two arms were matched for the number and duration of sessions. Participants in both arms were provided with eight weeks of nicotine patches. Gifford 2003 compared a seven‐week programme of individual and group ACT sessions (with no pharmacotherapy) with a lower‐intensity comparator group that received a seven‐week course of nicotine patches. O'Connor 2020 compared six weeks of face‐to‐face, group ACT sessions with six weeks of face‐to‐face, group behavioural support, matched in intensity to the six‐week, face‐to‐face ACT programme. No pharmacotherapy was provided.

One study tested the effectiveness of ACT delivered through a combination of face‐to‐face sessions and a smartphone app. In addition to the face‐to‐face‐only intervention arm, O'Connor 2020 included a second intervention arm in which ACT was delivered via two modalities: the six‐week, face‐to‐face ACT programme and an ACT‐based smartphone app. This combined ACT intervention was compared with a less intensive ACT arm (i.e. 6 weeks of face‐to‐face ACT without the app).

One study tested the effectiveness of ACT delivered exclusively via smartphone app. Bricker 2020 compared an ACT smartphone app with a smoking cessation app based on national clinical practice guidelines. No pharmacotherapy was provided.

One study tested the effectiveness of ACT delivered through a combination of face‐to‐face sessions and telephone calls. Mak 2020 compared ACT delivered in one face‐to‐face session and two follow‐up telephone calls with brief advice (5 minutes). Participants in both arms were also provided with self‐help materials. No pharmacotherapy was provided.

One study tested the effectiveness of ACT delivered exclusively via telephone. Bricker 2014a compared an ACT programme delivered over five telephone calls with standard quitline CBT. The arms were matched for the number and duration of telephone calls. Participants were provided with two weeks of nicotine patches or gum.

Two studies tested the effectiveness of ACT delivered via websites. Bricker 2018 compared an online ACT programme with a national standard online quit programme, with both arms receiving daily messages prompting them to log in. Savvides 2014 compared an avatar‐led, internet‐based ACT programme with a waitlist control. Neither study provided participants with pharmacotherapy.

BCTs varied across studies. The most commonly used techniques included problem solving (6 studies), body changes (5 studies), goal setting (4 studies), and action planning (4 studies), with no clear patterning in the number or type of BCTs used across mode of delivery.

###### Distress tolerance training

Two studies used distress tolerance training. Distress tolerance training interventions combined elements drawn from ACT with exposure‐based treatment. Exposure included periods of scheduled abstinence prior to sessions and exposure to cues within sessions, allowing ACT skills to be practised within the sessions in response to internal triggers.

Bloom 2020 targeted women who were concerned about post‐cessation weight gain. The intervention was nine weeks of CBT plus distress tolerance training ‐ a face‐to‐face and telephone programme that targeted the fear of anticipated post‐cessation weight gain and facilitated initiation of abstinence, and appetite awareness and mindful eating skills to reduce post‐cessation emotional eating. The comparator was nine weeks of CBT plus smoking health education, which mentioned diet and exercise as strategies for health promotion but did not specifically recommend changing diet or increasing physical activity to prevent post‐cessation weight gain.

Brown 2013 targeted smokers who had previously tried to quit but had never been able to remain abstinent for more than 72 hours. The intervention was eight weeks of face‐to‐face distress tolerance treatment and the comparator was six weeks of standard treatment.

Both studies also provided NRT (8 weeks of nicotine patches) to all participants in the intervention and comparator arms.

BCTs varied across studies: while both used pharmacological support, Brown 2013 used reduce prompts/cues and Bloom 2020 used problem solving, self‐monitoring of behaviour, social support, information about health consequences, and anticipated regret.

###### Yoga

Three studies used yoga involving a mindfulness‐based approach.

Two studies used Vinyasa yoga (Bock 2012; Gaskins 2015), and one used Iyengar yoga (Bock 2019). In each study, participants in the intervention arm were provided with eight CBT classes and 16 yoga classes over eight weeks. Participants in the comparator arm received CBT and wellness classes over eight weeks. In Bock 2012 and Bock 2019, the comparator was matched to the intervention in terms of the number and duration of wellness classes (16 x 1‐hour classes). However, in Gaskins 2015 the comparator was less intensive than the intervention: the intervention arm received 16 yoga classes over the eight weeks, each lasting 60 to 90 minutes, while the comparator arm received eight brief wellness discussions following the CBT sessions.

None of the studies provided participants with pharmacotherapy, but two studies (Bock 2012; Bock 2019), noted that participants were permitted to use NRT or other medications alongside the programme if they wanted to.

BCTs were similar across studies: all three studies used goal setting, problem solving, and social support. Bock 2012 and Gaskins 2015 also used self‐monitoring of behaviour and body changes, and Gaskins 2015 also used reduce negative emotions. 

##### Outcomes

###### Smoking abstinence

The included studies provided a range of smoking abstinence outcome measures. Two studies reported the strictest outcome as biochemically verified continuous abstinence (Bock 2019; Davis 2014a), 12 studies defined abstinence as biochemically verified, seven‐day point prevalence (Bloom 2020; Bock 2012; Brown 2013; Davis 2014b; Garrison 2020; Gaskins 2015; Gifford 2003; Mak 2020; O'Connor 2020; Pbert 2020; Vidrine 2016; Weng 2021), one study as biochemically verified, 30‐day point prevalence (McClure 2020), and one study as carbon monoxide less than 10 parts per million (de Souza 2020). 

Four additional studies reported self‐reported continuous abstinence (Bricker 2020), self‐reported seven‐day point prevalence (Singh 2014), or self‐reported 30‐day point prevalence (Bricker 2014a; Bricker 2018), without biochemical verification. 

Most studies had a maximum follow‐up duration of six months, but six studies collected their final follow‐up data at 12 months (Bricker 2018; Bricker 2020; Gifford 2003; Mak 2020; McClure 2020; Singh 2014). Savvides 2014 reported collecting data on seven‐day and 30‐day point prevalence abstinence at six and 12 months but at the time this report was published data collection was ongoing and the only smoking abstinence outcomes reported are from immediately post‐treatment; we have not been able to find long‐term outcome data reported elsewhere.

###### Mental health

Ten of the included studies reported collecting data on mental health and well‐being (Bloom 2020; Bock 2012; Brown 2013; Davis 2014a; Davis 2014b; de Souza 2020; Gaskins 2015; Gifford 2003; O'Connor 2020; Vidrine 2016), of which nine analysed and reported on changes in these outcomes. Mental health outcomes included depression, anxiety, perceived stress, and negative and positive affect. The constructs assessed, measures used, and follow‐up durations varied across studies.

#### Excluded studies

Figure 1 shows the most common reasons for exclusion of studies during full‐text screening, which included: a follow‐up period of less than six months; ineligible study design (not an RCT); conference proceedings with no relevant studies; and an intervention where it was not possible to isolate the effects of the mindfulness element.

In the Characteristics of excluded studies table, we list exclusion reasons for 47 studies. This list is not comprehensive, only containing studies that a reader might plausibly expect to be included.

### Risk of bias in included studies

Overall, we judged four of the 21 completed studies to be at low risk of bias, nine studies to be at unclear risk, and the remaining eight studies at high risk of bias.

Details of risk of bias judgments for each domain of each included study can be found in the Characteristics of included studies table. Figure 2 illustrates judgments for each included study.

**2:**
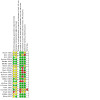
Figure 2: risk of bias summary

#### Allocation

##### Random sequence generation

We judged one study (Singh 2014), to be at high risk of selection bias for sequence generation, because randomisation was via alternate placement in the experimental and control groups. We judged eight studies at low risk of bias (Bock 2019; Bricker 2014a; Bricker 2018; Bricker 2020; Gifford 2003; Mak 2020; O'Connor 2020; Savvides 2014). The risk of bias for the remaining studies was unclear.

##### Allocation concealment

We judged six studies (Bricker 2014a; Bricker 2018; Bricker 2020; Mak 2020; O'Connor 2020; Weng 2021), to be at low risk of selection bias for allocation concealment, and the remainder to be at unclear risk as there was insufficient information with which to judge.

#### Blinding

##### Blinding of participants and personnel (performance bias)

As we were investigating a primarily behavioural intervention, we did not assess the blinding of participants and providers, as it is impossible to blind people to behavioural interventions. This is in accordance with specific guidance from the Cochrane Tobacco Addiction Group.

##### Blinding of outcome assessment (detection bias)

We rated three studies (Mak 2020; Savvides 2014; Singh 2014), at high risk for detection bias. These studies did not use blinding, they provided different levels of support, and outcomes were self‐reported. This meant we thought there was a high risk of bias being introduced. We judged the remaining studies to be at low risk for detection bias.

#### Incomplete outcome data

We judged most studies (13 out of 21) to be at low risk of attrition bias. We rated four studies with substantial (> 50%) loss to follow‐up and one study with more than a 20% difference in follow‐up rates between arms at high risk of attrition bias (Davis 2014a; Davis 2014b; de Souza 2020; Gaskins 2015; Mak 2020). The remaining three studies (Bock 2012; Savvides 2014; Singh 2014), did not provide sufficient data on which to judge, and hence we judged them to be at unclear risk.

#### Selective reporting

Of the 21 studies, we considered 13 to be at low risk of reporting bias, as they reported all prespecified or expected outcomes. We rated two studies (Bock 2012; de Souza 2020), at high risk, as they did not present data as specified in the original protocols. We judged the rest (Bloom 2020; Brown 2013; Gifford 2003; Mak 2020; Savvides 2014; Singh 2014), to be at unclear risk, as we were unable to identify a protocol.

#### Other potential sources of bias

We judged one study (Savvides 2014), to be at high risk of other bias because it used a waitlist control. This design risks participants in the control arm delaying quitting, knowing that they would be receiving an intervention at a later date. This has the potential to inflate the reported effect of the intervention. We did not find any other studies to be at risk of other bias.

### Effects of interventions

See: Table 1; Table 2; Table 3; Table 4

#### Mindfulness training 

##### Smoking abstinence

Three studies compared an intervention involving mindfulness training with an alternative smoking cessation treatment that was matched for intensity (Davis 2014b; Pbert 2020; Vidrine 2016). Pooled data showed no evidence of a benefit of mindfulness training, with a point estimate very close to the null (RR 0.99, 95% CI 0.67 to 1.46; I^2^ = 0%; 542 participants; low‐certainty evidence; Analysis 1.1). We judged Davis 2014b at high risk of bias, while the other two studies were at unclear risk. Removing the study at high risk of bias did not substantially change the interpretation of the results (RR 0.82, 95% CI 0.51 to 1.33; I^2^ = 0%; 2 studies, 407 participants; Analysis 5.1), nor did using complete‐case analysis (RR 1.05, 95% CI 0.69 to 1.59; I^2^ = 25%; 3 studies, 356 participants; Analysis 6.1). In each of these sensitivity analyses, there was only a minimal impact on the point estimate, with CIs still spanning both benefit (i.e. higher quit rates) and harm (i.e. lower quit rates). A subgroup analysis separating studies by mode of delivery showed no evidence of moderating the effect of mindfulness training interventions in comparison with alternative, matched‐intensity smoking cessation treatment (I^2^ = 0%). Pbert 2020 was a cluster‐RCT, so we conducted another sensitivity analysis adjusting for any potential clustering effect (assuming an ICC of 0.03); this did not substantially change the overall pooled result (RR 1.01, 95% CI 0.68 to 1.50; I^2^ = 0%; Analysis 7.1).

Five studies compared an intervention involving mindfulness training with a less intensive smoking cessation treatment (Davis 2014a; Pbert 2020; Singh 2014; Vidrine 2016; Weng 2021). Pooled data showed no evidence of a benefit of mindfulness training, with the CI spanning both benefit and harm of mindfulness training interventions in comparison with less intensive smoking cessation treatments (RR 1.19, 95% CI 0.65 to 2.19; I^2^ = 60%; 813 participants; very low‐certainty evidence; Analysis 1.2). We judged Davis 2014a and Singh 2014 at high risk of bias, while the other three studies were at unclear risk. Removing the studies at high risk of bias changed the direction of the effect estimate (from favouring mindfulness training to favouring the comparator) but the CI still spanned both benefit and harm so this did not substantially change the interpretation of the results (RR 0.81, 95% CI 0.50 to 1.33; I^2^ = 5%; 3 studies, 566 participants; Analysis 5.2). Using complete‐case analysis produced similar results to the main analysis (RR 1.08, 95% CI 0.53 to 2.16; I^2^ = 62%; 4 studies, 479 participants; Analysis 6.2). Subgroup analyses showed some evidence of moderation by type of comparator (I^2^ = 68%). While there was no evidence of a benefit of mindfulness training versus less intensive behavioural support (RR 1.31, 95% CI 0.75 to 2.30; I^2^ = 60%; 2 studies, 453 participants), brief advice (RR 0.47, 95% CI 0.18 to 1.23; 1 study, 213 participants), or self‐help materials (RR 0.75, 95% CI 0.28 to 2.00; 1 study, 96 participants), there was evidence of a benefit of mindfulness training versus mixed treatment (treatment as usual, which varied between participants and encompassed a range of treatments such as behaviour therapies, NRT, and other medications; RR 2.77, 95% CI 1.30 to 5.94; 1 study, 51 participants). However, we judged the latter study at high risk of bias and it had substantial imprecision. Adjusting for clustering in Pbert 2020 (assuming an ICC of 0.03) did not substantially change the overall pooled result (RR 1.22, 95% CI 0.66 to 2.26; I^2^ = 58%; Analysis 7.2) or the subgroup result for mindfulness training versus self‐help materials (RR 0.80, 95% CI 0.24 to 2.67).

One study compared an intervention involving mindfulness training with no treatment. Garrison 2020 showed no evidence of a benefit of mindfulness training, with the point estimate favouring no treatment over mindfulness training and the CI spanning both benefit and harm of mindfulness training compared with no treatment (RR 0.81, 95% CI 0.43 to 1.53; 325 participants; low‐certainty evidence; Analysis 1.3). We judged this study at unclear risk of bias. Using complete‐case analysis did not substantially change the interpretation of the results (RR 0.77, 95% CI 0.41 to 1.43; 247 participants; Analysis 6.3).

One study compared an intervention involving mindfulness‐based relapse prevention with no treatment. de Souza 2020's point estimate favoured mindfulness‐based relapse prevention over no treatment but there was substantial imprecision, meaning the result could indicate potential harm as well as considerable benefit (RR 1.43, 95% CI 0.56 to 3.67; 86 participants; very low‐certainty evidence; Analysis 1.4). We judged this study at high risk of bias. Using complete‐case analysis did not substantially change the interpretation of the results (RR 1.23, 95% CI 0.72 to 2.10; 20 participants; Analysis 6.4).

##### Mental health

Three studies that tested an intervention involving mindfulness training reported on mental health outcomes (Analysis 1.5; very low‐certainty evidence). One study showed evidence of a benefit of mindfulness training on mental health. Davis 2014b (135 participants) analysed perceived stress at six months post‐quit. They observed a statistically significantly greater reduction in perceived stress between baseline and six months in the intervention arm than the intensity‐matched control arm, but this difference was not statistically significant when analysed as intention‐to‐treat.

Two studies showed no clear evidence of a benefit of mindfulness training on mental health. de Souza 2020 (86 participants) analysed depression, anxiety, negative affect, and positive affect at 4 and 12 weeks. No statistically significant or clinically meaningful difference between conditions was observed for any outcome at either time point. Vidrine 2016 (412 participants) assessed depression, perceived stress, negative affect, and positive affect at six time points between quit date and six months post‐quit. They analysed changes between quit date and six months and observed no statistically significant or clinically meaningful difference between conditions for any outcome.

In addition, Davis 2014a (196 participants) assessed negative affect at one month post‐baseline, but only reported data for the intervention arm.

#### Acceptance and commitment therapy (ACT)

##### Smoking abstinence

Five studies compared an intervention involving ACT with an alternative smoking cessation treatment that was matched for intensity (Bricker 2014a; Bricker 2018; Bricker 2020; McClure 2020; O'Connor 2020). We judged McClure 2020 to be at unclear risk of bias, while the other four studies were at low risk. It was not appropriate to pool data across these five studies because there was a high level of heterogeneity (I^2^ = 82%; Analysis 2.1), with variation in the direction of effect between studies, and the result may be misleading. Subgroup analyses showed some evidence of moderation by mode of delivery (I^2^ = 82%), although this didn't account for all variation within subgroups. While there was no evidence of a benefit of ACT delivered face‐to‐face (RR 0.76, 95% CI 0.32 to 1.78; I^2^ = 68%; 2 studies, 550 participants), by telephone (RR 1.35, 95% CI 0.74 to 2.46; 1 study, 121 participants), or via a website (RR 0.91, 95% CI 0.79 to 1.05; 1 study, 2637 participants), there was evidence of a benefit of ACT delivered via smartphone app (RR 1.77, 95% CI 1.32 to 2.37; 1 study, 2415 participants). Using complete‐case analysis did not substantially change the interpretation of results.

One study compared an intervention involving ACT with NRT. Gifford 2003's point estimate favoured ACT over NRT but there was substantial imprecision, meaning the result could indicate potential harm as well as considerable benefit (RR 1.27, 95% CI 0.53 to 3.02; 102 participants; low‐certainty evidence; Analysis 2.2). We judged this study to be at unclear risk of bias. Using complete‐case analysis increased the point estimate but did not substantially change the interpretation of the results (RR 1.63, 95% CI 0.71 to 3.72; 71 participants; Analysis 6.6).

One study compared an intervention involving ACT with brief advice. Mak 2020's point estimate favoured ACT over brief advice but there was substantial imprecision, meaning the result could indicate potential harm as well as considerable benefit (RR 1.27, 95% CI 0.59 to 2.75; 144 participants; very low‐certainty evidence; Analysis 2.3). We judged this study to be at high risk of bias. Using complete‐case analysis reduced the point estimate but did not substantially change the interpretation of the result (RR 1.06, 95% CI 0.54 to 2.11; 66 participants; Analysis 6.7).

One study compared an intervention involving ACT with less intensive ACT. O'Connor 2020 showed no evidence of a benefit of more intensive ACT, with the point estimate indicating no difference between more and less intensive ACT (RR 1.00, 95% CI 0.50 to 2.01; 100 participants; low‐certainty evidence; Analysis 2.4). We judged this study to be at low risk of bias. Using complete‐case analysis did not substantially change the interpretation of the result (RR 0.94, 95% CI 0.47 to 1.86; 91 participants; Analysis 6.8).

###### Within‐study analyses of moderators of interest

Bricker 2018 tested for moderation of the effectiveness of ACT by baseline mental health (depression or anxiety) and commitment to quitting. Quit rates were not found to differ significantly according to these variables.

Gifford 2003 tested the effectiveness of ACT in a subsample of smokers who were highly dependent. While there was no significant difference between the ACT arm and comparator arm in the full sample, ACT was reported to be associated with better long‐term quitting outcomes among nicotine‐dependent participants.

##### Mental health

Two studies that tested an intervention involving ACT reported on mental health outcomes (Analysis 2.5; very low‐certainty evidence). Both studies showed no evidence of a benefit of ACT on mental health. Gifford 2003 (102 participants) analysed negative affect between conditions at post‐treatment, six months and 12 months and O'Connor 2020 (150 participants) analysed positive mental health at post‐treatment and six months. Neither study observed a statistically significant or clinically meaningful difference between conditions at any time point.

#### Distress tolerance training interventions

##### Smoking abstinence

One study compared an intervention involving distress tolerance training with an alternative smoking cessation treatment that was matched for intensity. Bloom 2020 showed no evidence of a benefit of distress tolerance training, with the 95% CI spanning both benefit and harm (RR 0.87, 95% CI 0.26 to 2.98; 69 participants; low‐certainty evidence; Analysis 3.1). We judged this study to be at unclear risk of bias. Using complete‐case analysis did not substantially change interpretation of the result (RR 0.86, 95% CI 0.26 to 2.86; 54 participants; Analysis 6.9).

One study compared a distress tolerance training intervention with a less intensive smoking cessation treatment (Brown 2013). There was substantial imprecision, meaning the result could indicate potential harm as well as considerable benefit (RR 1.63, 95% CI 0.33 to 8.08; 49 participants; low‐certainty evidence; Analysis 3.2). We judged this study to be at unclear risk of bias. Using complete‐case analysis did not substantially change the interpretation of the result (RR 1.68, 95% CI 0.34 to 8.28; 46 participants; Analysis 6.10).

##### Mental health

One study that tested an intervention involving distress tolerance training reported on a mental health outcome (Analysis 3.3; low‐certainty evidence). Brown 2013 (49 participants) analysed negative affect at four weeks post‐quit and observed no statistically significant or clinically meaningful difference between conditions. 

In addition, Bloom 2020 (69 participants) planned to assess depression and negative affect at each follow‐up (1 month, 3 months, and 6 months post‐treatment), but to our knowledge have not reported these data.

#### Yoga

##### Smoking abstinence

One study compared an intervention involving yoga with an alternative smoking cessation treatment that was matched for intensity. Bock 2012's point estimate favoured yoga over alternative smoking cessation treatment but there was substantial imprecision, meaning the result could indicate potential harm as well as considerable benefit (RR 1.44, 95% CI 0.40 to 5.16; 55 participants; very low‐certainty evidence; Analysis 4.1). We judged this study to be at high risk of bias. The number of participants followed up in each arm was unclear so we could not conduct a complete‐case analysis.

Raw data on the number of quits at six months were not available for two other studies that tested an intervention involving yoga so we could not calculate unadjusted RRs. Bock 2019 reported no significant difference in the odds of smoking abstinence between the intervention arm and matched comparator arm at six‐month follow‐up (P > 0.05). Gaskins 2015 also reported no significant difference in the odds of smoking abstinence between the intervention arm and less intensive comparator arm at six‐month follow‐up (OR 2.38, 95% CI 0.52 to 10.8, P = 0.265). There was substantial imprecision, meaning the result could indicate potential harm as well as considerable benefit.

##### Mental health

Two studies that tested a yoga intervention reported on mental health outcomes (Analysis 4.2). Both studies showed no evidence of a benefit of yoga on mental health. Bock 2012 (55 participants) analysed depression, anxiety, and general well‐being at eight weeks (post‐treatment) and six months, but only reported data collected at 8 weeks. No statistically significant or clinically meaningful differences between conditions were observed. Gaskins 2015 (38 participants) analysed depression, anxiety, and a composite measure of physical self‐worth, attractiveness, physical strength, and condition, at eight weeks (post‐treatment), three months and six months. No statistically significant or clinically meaningful differences between conditions, nor any significant group by time interactions, were observed.

## Discussion

### Summary of main results

The 21 studies in this review did not detect a clear, long‐term benefit of mindfulness‐based smoking cessation interventions (based on mindfulness training, ACT, distress tolerance training, or yoga) when compared with other interventions, or with no intervention, for smoking cessation. This was true when mindfulness‐based interventions were compared with intensity‐matched smoking cessation interventions, less intensive smoking cessation interventions (including less intensive mindfulness), or no treatment. However, one subgroup analysis found a positive effect of an ACT intervention when this was delivered via smartphone application, as opposed to face‐to‐face, through a website, or over the telephone. 

Ten studies collected data on mental health and well‐being, of which nine analysed and reported on changes in these outcomes. There was no clear evidence of a positive or negative effect of mindfulness‐based treatments on mental health and well‐being.

### Overall completeness and applicability of evidence

The searches conducted for this review were broad, in our attempt to find any study that made any mention of mindfulness‐based approaches. As well as medical databases, we also searched studies registers to identify any ongoing or completed but unpublished registered studies, and supplemented our traditional search strategy with an automated search strategy developed as part of the Human Behaviour Change Project (Michie 2017), using Microsoft Academic. We therefore feel confident in our search approach.

A particular challenge of this review compared with other reviews of smoking cessation treatments was bringing together a diverse evidence base. The studies identified by this review varied widely in their design (e.g. digital versus in person), intervention type (e.g. ACT versus yoga), nature of the comparator, and mental health outcomes assessed, meaning we could not meaningfully pool results in a single meta‐analysis. While we intentionally adopted an inclusive approach to cover a broad range of mindfulness‐based interventions, some of the studies included may have had a looser mindfulness focus (e.g. yoga interventions) than others (e.g. mindfulness training interventions), but our approach to pooling meant that we did not 'dilute' the effects of pure mindfulness interventions. The studies identified in this review were mainly conducted in the USA and all took place in high‐income or higher middle‐income countries. Most studies were carried out in the general population and so may not be applicable to populations with specific requirements or particularly high cigarette dependence.

To be included studies had to assess long‐term abstinence, so most studies were able to contribute cessation data to the relevant comparisons. However, the number of studies and participants contributing to each analysis were low, and further research could strengthen or change findings. In addition, data on mental health outcomes were sparse and varied, meaning we were unable to conduct meta‐analyses for this outcome. We did not assess safety outcomes beyond any adverse effect on mental health and well‐being because the intervention was behavioural and was not considered high risk for adverse events.

### Quality of the evidence

Of the 21 studies included in this review, we judged four to be at low risk of bias for all domains, and eight to be at high risk in one or more domains. In many cases, we rated studies at unclear risk of bias because they did not report key information. In these cases, it is impossible to know whether these studies were at any risk of bias or whether the information was simply not reported. To investigate the potential impact on results of studies that we judged to be at high risk of bias, we removed these studies in sensitivity analyses. This did not affect our interpretation of results.

We considered the certainty of the evidence for effectiveness of mindfulness training, ACT, distress tolerance training, and yoga interventions for smoking cessation relative to matched‐intensity smoking cessation treatment, less intensive smoking cessation treatment, or no treatment. We created summary of findings tables and carried out GRADE ratings for each comparison (Table 1; Table 2; Table 3; Table 4).

We judged all comparisons and outcomes to be of low or very low certainty, meaning that the interpretation of effects is likely to change as more studies and information become available. Reasons for downgrading the certainty of evidence included: risk of bias, when all studies pooled were judged at high risk of bias; inconsistency, when there was variance in the characteristics of studies or statistical heterogeneity was high and unexplained; and imprecision, when the absolute number of events was low or confidence intervals were wide and included no difference, or both.

### Potential biases in the review process

We took several steps to ensure the review process was robust. We followed standard methods used by the Cochrane Tobacco Addiction Group. Our search strategy included a broad range of databases, including the Cochrane Tobacco Addiction Group's Specialised Register. We followed standard Cochrane practice of requiring two review authors to independently screen studies, extract data, and assess risk of bias. None of the authors of this review were also authors of included studies.

Despite this rigorous approach, it is possible that relevant literature, particularly unpublished or grey literature, may have been missed. We did not evaluate publication bias as there were fewer than 10 studies available for each primary outcome. It is also possible that non‐reporting of information in the published articles may have influenced the risk of bias assessments.

### Agreements and disagreements with other studies or reviews

Carim‐Todd 2013 conducted a systematic review of yoga and other mind‐body interventions for smoking cessation. It included 14 studies, of which eight studies were RCTs, one study was a non‐RCT, two studies applied within‐participant controlled designs, and three studies used pre‐post designs. We included just one of these RCTs in our review (Bock 2012); the others did not meet our inclusion criterion of six months' follow‐up. Carim‐Todd 2013 did not meta‐analyse data due to differences in study designs, participants, and outcome measures. The authors reported that all 14 included studies, "observed changes in smoking behavior or in predictors of smoking behavior that could be beneficial for smoking cessation" but more clinical studies with larger sample sizes and carefully monitored interventions were required to draw firm conclusions.

Maglione 2017 conducted a meta‐analysis of mindfulness meditation interventions for smoking cessation. It included 10 RCTs: four mindfulness training studies that we included in the current review (Davis 2014a; Davis 2014b; Singh 2014; Vidrine 2016), and six studies that did not meet our inclusion criterion of six months' follow‐up. Pooled data from six of the 10 studies included by Maglione 2017 showed no significant effect of mindfulness meditation versus comparator interventions (odds ratio 2.52, 95% CI 0.76 to 8.29; I^2^ = 58%; 936 participants). This is consistent with our results.

Oikonomou 2017 conducted a meta‐analysis of mindfulness training interventions for smoking cessation compared with current behavioural treatments (the Freedom From Smoking programme and smoking quit line). It included four RCTs: two mindfulness training studies we included in the current review (Davis 2014a; Davis 2014b) and two studies that did not meet our inclusion criterion of six months' follow‐up. Pooled data from three of their four included studies showed significantly greater smoking abstinence rates in smokers who received mindfulness treatment compared to control at 17‐ to 24‐week follow‐up (RR 1.88, 95% CI 1.04 to 3.40; I^2^ = 44%; 419 participants). This differs from our results. The two studies in this analysis that we included in our review showed no benefit of mindfulness training (Davis 2014a; Davis 2014b). We excluded the third from our review because its longest follow‐up was just 17 weeks (Brewer 2011). Brewer 2011 accounts for the difference between the conclusions of Oikonomou 2017's review and our review. Brewer 2011's results indicated a strong benefit of mindfulness training, albeit with high levels of imprecision (RR 4.97, 95% CI 1.52 to 16.22; 88 participants).

Goldberg 2018 conducted a meta‐analysis of mindfulness‐based interventions for psychiatric disorders, including smoking as a form of substance use. It included seven RCTs that focused on smoking cessation, of which four compared a mindfulness‐based intervention with evidence‐based treatment. Three of these studies were included in the current review (Davis 2014a; Davis 2014b; Vidrine 2016), but one did not meet our inclusion criterion of six months' follow‐up. Pooled data from four of the seven included studies showed significantly greater smoking abstinence rates in smokers who received mindfulness treatment compared to control (*d* 0.42, 95% CI 0.20 to 0.64; I^2^ = 11%; 587 participants). Similar to Oikonomou 2017, this differs from our results because it included Brewer 2011, which showed a strong benefit of mindfulness on abstinence rates.

## Authors' conclusions

**Implications for practice**

- We did not detect a clear benefit of mindfulness‐based interventions for increasing long‐term smoking quit rates compared with no treatment or alternative smoking cessation treatments that are equally or less intensive. However this evidence is of low or very low certainty, and further evidence is likely to change our conclusions.
- We also did not detect a clear benefit of mindfulness‐based interventions for improving mental health and well‐being compared with no treatment or alternative smoking cessation treatments that are equally or less intensive. Again, this evidence is of low or very low certainty, and our conclusions are likely to change with further evidence.

**Implications for research**

- Further RCTs of mindfulness‐based interventions for smoking cessation are needed, following up participants at six months or longer. Studies with active comparators (i.e. comparing mindfulness‐based interventions to currently used smoking cessation interventions) are likely to be of particular use to decision makers. 
- Further studies need to be adequately powered to detect potentially small but clinically important differences between mindfulness‐based interventions and active comparators. In order to ensure low risk of bias, they should involve biochemical verification of abstinence along with improved methods of retaining participants to follow‐up points. 
- There is also a need for more consistent reporting of mental health and well‐being outcomes in studies of mindfulness‐based interventions for smoking cessation. Even if mindfulness is only as successful as other behavioural support in enhancing long‐term quit rates, it may be preferable to some smokers if it improves mental health. Most studies we identified did not report on mental health or well‐being. Those that did assessed a number of different constructs, at different time points, using a variety of measures, meaning we could not meaningfully pool the results. Therefore, it would be useful to develop a consensus on the best ways to measure these outcomes in relevant studies.

## History

Protocol first published: Issue 7, 2020

## Appendices

### Appendix 1. Search strategies

**Cochrane Tobacco Addiction Group Specialised Register**

1. MESH DESCRIPTOR Mindfulness EXPLODE ALL 
2. MESH DESCRIPTOR Meditation EXPLODE ALL 
3. MESH DESCRIPTOR Mind‐Body Therapies EXPLODE ALL 
4. MESH DESCRIPTOR Mind‐Body Relations, Metaphysical EXPLODE ALL 
5. MESH DESCRIPTOR Breathing Exercises EXPLODE ALL 
6.  ((meditat* OR mindful* OR relaxation* mind‐body OR body‐mind)):TI,AB,MH,EMT,KY,XKY 
7.  ((Samadhi OR Samapatti)):TI,AB,MH,EMT,KY,XKY 
8.  ((acceptance adj2 commitment)):TI,AB,MH,EMT,KY,XKY 
9. #8 OR #7 OR #6 OR #5 OR #4 OR #3 OR #2 OR #1 

**CENTRAL**

1. MESH DESCRIPTOR Mindfulness EXPLODE ALL 
2. MESH DESCRIPTOR Meditation EXPLODE ALL 
3. MESH DESCRIPTOR Mind‐Body Therapies EXPLODE ALL 
4. MESH DESCRIPTOR Mind‐Body Relations, Metaphysical EXPLODE ALL
5. MESH DESCRIPTOR Breathing Exercises EXPLODE ALL 
6.  ((meditat* OR mindful* OR relaxation* mind‐body OR body‐mind)):TI,AB,MH,EMT,KY,XKY 
7.  ((Samadhi OR Samapatti)):TI,AB,MH,EMT,KY,XKY 
8.  ((acceptance adj2 commitment)):TI,AB,MH,EMT,KY,XKY 
9. #8 OR #7 OR #6 OR #5 OR #4 OR #3 OR #2 OR #1 
10. MESH DESCRIPTOR Tobacco Use Disorder EXPLODE ALL NOT SRTAG 
11. MESH DESCRIPTOR Tobacco Use Cessation EXPLODE ALL NOT SRTAG 
12. MESH DESCRIPTOR Tobacco Smoke Pollution EXPLODE ALL NOT SRTAG 
13. MESH DESCRIPTOR Tobacco Use Cessation Products EXPLODE ALL NOT SRTAG 
14. MESH DESCRIPTOR Tobacco, Smokeless EXPLODE ALL NOT SRTAG 
15.  (SMOKING* or TOBACCO or TOBACCO‐USE‐DISORDER* or TOBACCO‐SMOKELESS* or TOBACCO‐SMOKE‐POLLUTION* or TOBACCO‐USE‐CESSATION* or NICOTINE*):MH NOT SRTAG 
16.  (smoking cessation):MH NOT SRTAG 
17.  (SMOKING CESSATION or ANTISMOK*):TI,AB NOT SRTAG 
18.  (quit* or smok* or nonsmok* or cigar* or tobacco* or nicotine*):TI NOT SRTAG
19. MESH DESCRIPTOR Smoking Cessation EXPLODE ALL NOT SRTAG 
20. #10 OR #11 OR #12 OR #13 OR #14 OR #15 OR #16 OR #17 OR #18 OR #19
21. #20 AND #9 

**MEDLINE/Embase/PsycINFO** (in Ovid)

1. exp Smoking Cessation/ 
2. exp "Tobacco Use Disorder"/ 
3.  (SMOKING* or TOBACCO or "TOBACCO USE DISORDER*" or "TOBACCO USE CESSATION*" or NICOTINE*).mp. 
4.  (SMOKING CESSATION or ANTISMOK*).ti,ab. 
5.  (quit* or smok* or nonsmok* or cigar* or tobacco* or nicotine*).ti. 
6. smoking cessation.mp. 
7. exp "Tobacco Use Cessation"/ 
8. 1 or 2 or 3 or 4 or 5 or 6 or 7 
9. exp mindfulness/ 
10. exp meditation/ 
11. exp "Mind Body Therapies"/ 
12. exp "Mind Body Relations, Metaphysical"/ 
13. exp Breathing Exercises/ 
14.  (meditat* or mindful* or "relaxation* mind body" or "body mind").mp. 
15.  (Samadhi or Samapatti).mp. 
16.  (acceptance adj2 commitment).ti,ab. 
17. 9 or 10 or 11 or 12 or 13 or 14 or 15 or 16 
18. 8 and 17

## Data and analyses

**Comparison 1:** Mindfulness training

Outcome or subgroup title	No. of studies	No. of participants	Statistical method	Effect size
1.1 Mindfulness training vs matched‐intensity smoking cessation treatment	3	542	Risk Ratio (M‐H, Random, 95% CI)	0.99 [0.67, 1.46]
1.1.1 Face‐to‐face	2	444	Risk Ratio (M‐H, Random, 95% CI)	1.05 [0.64, 1.72]
1.1.2 Smartphone app	1	98	Risk Ratio (M‐H, Random, 95% CI)	0.78 [0.29, 2.08]
1.2 Mindfulness training vs less intensive smoking cessation treatment	5	813	Risk Ratio (M‐H, Random, 95% CI)	1.19 [0.65, 2.19]
1.2.1 vs. less intensive behavioural support	2	453	Risk Ratio (M‐H, Random, 95% CI)	1.31 [0.75, 2.30]
1.2.2 vs. brief advice	1	213	Risk Ratio (M‐H, Random, 95% CI)	0.47 [0.18, 1.23]
1.2.3 vs. self‐help materials	1	96	Risk Ratio (M‐H, Random, 95% CI)	0.75 [0.28, 2.00]
1.2.4 vs. mixed treatment	1	51	Risk Ratio (M‐H, Random, 95% CI)	2.77 [1.30, 5.94]
1.3 Mindfulness training vs no treatment	1	325	Risk Ratio (M‐H, Random, 95% CI)	0.81 [0.43, 1.53]
1.4 Mindfulness‐based relapse prevention vs no treatment	1	86	Risk Ratio (M‐H, Random, 95% CI)	1.43 [0.56, 3.67]
1.5 Mental health outcomes	4		Other data	No numeric data

**Comparison 2:** Acceptance and commitment therapy (ACT)

Outcome or subgroup title	No. of studies	No. of participants	Statistical method	Effect size
2.1 ACT vs matched‐intensity smoking cessation treatment	5		Risk Ratio (M‐H, Random, 95% CI)	Subtotals only
2.1.1 Face‐to‐face	2	550	Risk Ratio (M‐H, Random, 95% CI)	0.76 [0.32, 1.78]
2.1.2 Telephone	1	121	Risk Ratio (M‐H, Random, 95% CI)	1.35 [0.74, 2.46]
2.1.3 Website	1	2637	Risk Ratio (M‐H, Random, 95% CI)	0.91 [0.79, 1.05]
2.1.4 Smartphone app	1	2415	Risk Ratio (M‐H, Random, 95% CI)	1.77 [1.32, 2.37]
2.2 ACT vs NRT	1	102	Risk Ratio (M‐H, Random, 95% CI)	1.27 [0.53, 3.02]
2.3 ACT vs brief advice	1	144	Risk Ratio (M‐H, Random, 95% CI)	1.27 [0.59, 2.75]
2.4 ACT vs less intensive ACT	1	100	Risk Ratio (M‐H, Random, 95% CI)	1.00 [0.50, 2.01]
2.5 Mental health outcomes	2		Other data	No numeric data

**Comparison 3:** Distress tolerance

Outcome or subgroup title	No. of studies	No. of participants	Statistical method	Effect size
3.1 Distress tolerance vs matched‐intensity smoking cessation treatment	1	69	Risk Ratio (M‐H, Random, 95% CI)	0.87 [0.26, 2.98]
3.2 Distress tolerance vs less intensive smoking cessation treatment	1	49	Risk Ratio (M‐H, Random, 95% CI)	1.63 [0.33, 8.08]
3.3 Mental health outcomes	2		Other data	No numeric data

**Comparison 4:** Yoga

Outcome or subgroup title	No. of studies	No. of participants	Statistical method	Effect size
4.1 Yoga vs matched‐intensity smoking cessation treatment	1	55	Risk Ratio (M‐H, Random, 95% CI)	1.44 [0.40, 5.16]
4.2 Mental health outcomes	2		Other data	No numeric data

**Comparison 5:** Sensitivity analysis ‐ excluding studies at high risk of bias

Outcome or subgroup title	No. of studies	No. of participants	Statistical method	Effect size
5.1 Mindfulness training vs matched‐intensity smoking cessation treatment	2	407	Risk Ratio (M‐H, Random, 95% CI)	0.82 [0.51, 1.33]
5.2 Mindfulness training vs less intensive smoking cessation treatment	3	566	Risk Ratio (M‐H, Random, 95% CI)	0.81 [0.50, 1.33]
5.2.1 vs. less intensive behavioural support	1	257	Risk Ratio (M‐H, Random, 95% CI)	1.11 [0.57, 2.18]
5.2.2 vs. brief advice	1	213	Risk Ratio (M‐H, Random, 95% CI)	0.47 [0.18, 1.23]
5.2.3 vs. self‐help materials	1	96	Risk Ratio (M‐H, Random, 95% CI)	0.75 [0.28, 2.00]

**Comparison 6:** Sensitivity analysis ‐ complete cases

Outcome or subgroup title	No. of studies	No. of participants	Statistical method	Effect size
6.1 Mindfulness training vs matched‐intensity smoking cessation treatment	3	356	Risk Ratio (M‐H, Random, 95% CI)	1.05 [0.69, 1.59]
6.2 Mindfulness training vs less intensive smoking cessation treatment	4	479	Risk Ratio (M‐H, Random, 95% CI)	1.08 [0.53, 2.16]
6.2.1 vs. less intensive behavioural support	2	224	Risk Ratio (M‐H, Random, 95% CI)	1.72 [0.62, 4.80]
6.2.2 vs. brief advice	1	165	Risk Ratio (M‐H, Random, 95% CI)	0.50 [0.19, 1.29]
6.2.3 vs. self‐help materials	1	90	Risk Ratio (M‐H, Random, 95% CI)	0.78 [0.30, 2.08]
6.3 Mindfulness training vs no treatment	1	247	Risk Ratio (M‐H, Random, 95% CI)	0.77 [0.41, 1.43]
6.4 Mindfulness‐based relapse prevention vs no treatment	1	20	Risk Ratio (M‐H, Random, 95% CI)	1.23 [0.72, 2.10]
6.5 ACT vs matched‐intensity smoking cessation treatment	5	4537	Risk Ratio (M‐H, Random, 95% CI)	1.05 [0.70, 1.57]
6.5.1 Face‐to‐face	2	437	Risk Ratio (M‐H, Random, 95% CI)	0.71 [0.35, 1.44]
6.5.2 Telephone	1	81	Risk Ratio (M‐H, Random, 95% CI)	1.14 [0.66, 1.96]
6.5.3 Website	1	2309	Risk Ratio (M‐H, Random, 95% CI)	0.93 [0.81, 1.07]
6.5.4 Smartphone app	1	1710	Risk Ratio (M‐H, Random, 95% CI)	1.85 [1.39, 2.47]
6.6 ACT vs NRT	1	71	Risk Ratio (M‐H, Random, 95% CI)	1.63 [0.71, 3.72]
6.7 ACT vs brief advice	1	66	Risk Ratio (M‐H, Random, 95% CI)	1.06 [0.54, 2.11]
6.8 ACT vs less intensive ACT	1	91	Risk Ratio (M‐H, Random, 95% CI)	0.94 [0.47, 1.86]
6.9 Distress tolerance vs matched‐intensity smoking cessation treatment	1	54	Risk Ratio (M‐H, Random, 95% CI)	0.86 [0.26, 2.86]
6.10 Distress tolerance vs less intensive smoking cessation treatment	1	46	Risk Ratio (M‐H, Random, 95% CI)	1.68 [0.34, 8.28]

**Comparison 7:** Sensitivity analysis ‐ mindfulness training adjusting for clustering in Pbert 2020

Outcome or subgroup title	No. of studies	No. of participants	Statistical method	Effect size
7.1 Mindfulness training vs matched‐intensity smoking cessation treatment	3	501	Risk Ratio (M‐H, Random, 95% CI)	1.01 [0.68, 1.50]
7.1.1 Face‐to‐face	2	444	Risk Ratio (M‐H, Random, 95% CI)	1.05 [0.64, 1.72]
7.1.2 Smartphone app	1	57	Risk Ratio (M‐H, Random, 95% CI)	0.83 [0.25, 2.77]
7.2 Mindfulness training vs less intensive smoking cessation treatment	5	773	Risk Ratio (M‐H, Random, 95% CI)	1.22 [0.66, 2.26]
7.2.1 vs. less intensive behavioural support	2	453	Risk Ratio (M‐H, Random, 95% CI)	1.31 [0.75, 2.30]
7.2.2 vs. brief advice	1	213	Risk Ratio (M‐H, Random, 95% CI)	0.47 [0.18, 1.23]
7.2.3 vs. self‐help materials	1	56	Risk Ratio (M‐H, Random, 95% CI)	0.80 [0.24, 2.67]
7.2.4 vs. mixed treatment	1	51	Risk Ratio (M‐H, Random, 95% CI)	2.77 [1.30, 5.94]

## Characteristics of studies

### Characteristics of included studies [ordered by study ID]

**Table:** Bloom 2020

***Study characteristics***		
Methods	**Study design:** RCT **Location:** USA **Setting:** community **Recruitment:** paper and electronic advertisements **Study dates:** not reported	
Participants	N = 69 **Specialist population?:** women motivated to quit smoking and concerned about post‐cessation weight gain **Definition of smoker used:** ≥ 5 combustible cpd for at least the past year **Participant characteristics:** 100% female; average age: 50 years; 74% white; 42% SES; average cpd: 16; nicotine dependence: average FTND 4.8	
Interventions	All participants received CBT, including preparation for quit date, reinforcement, and support for quitting, discussion of past and ongoing quit experiences, initiation of self‐monitoring, identification of triggers and high‐risk situations, development of coping strategies for triggers, obtaining social support, instruction in how to use nicotine patches, and relapse prevention. Recommendations for how to minimise weight gain were brief, de‐emphasised, and consistent with standard CBT (e.g. take a walk to cope with craving instead of smoking, eat low‐calorie snacks) **Comparator** CBT + smoking health education. Diet and exercise were mentioned as strategies for health promotion and prevention of disease but no specific recommendations for how to change diet or increase physical activity **Mode of delivery:** face‐to‐face (individual and group), telephone **Intensity:** 9 sessions (1 x 60 min, 8 x 90 min) and 1 phone call (20 min) over 9 weeks **Pharmacotherapy:** 8 weeks of nicotine patches from quit date, adjusted to individual cpd Type of therapist/provider: each session was co‐led by 2 trained group leaders, including a physician, doctoral‐level psychologists, and clinical psychology doctoral students **BCTs:** 1.2 Problem solving; 2.3 Self‐monitoring of behaviour; 3.1 Social support; 5.1 Information about health consequences; 11.1 Pharmacological support **Intervention** CBT + distress tolerance for weight concern. Based on ACT and included: psychoeducation about the relationship between smoking and weight; distress tolerance skills; discussion of weight concern as a barrier to successful initiation of abstinence; and values‐oriented living skills targeting reduction of emotional eating after quitting **Mode of delivery:** face‐to‐face (individual and group), telephone **Intensity:** 9 sessions (1 x 60 min, 8 x 90 min) and 1 phone call (20 min) over 9 weeks **Pharmacotherapy:** 8 weeks of nicotine patches from quit date, adjusted to individual cpd **Type of therapist/provider:** each session was co‐led by 2 trained group leaders, including a physician, doctoral‐level psychologists, and clinical psychology doctoral students **BCTs:** 1.2 Problem solving; 2.3 Self‐monitoring of behaviour; 3.1 Social support; 5.1 Information about health consequences; 5.5 Anticipated regret; 11.1 Pharmacological support	
Outcomes	**Definition of abstinence:** 7‐day point prevalence **Longest follow‐up:** 6 months **Biochemical verification:** CO ≤ 6 ppm **Other relevant outcomes reported:** negative affect	
Notes	**Relevant comparisons:** distress tolerance for weight concern + CBT + NRT vs health education + CBT + NRT **Funding source:** National Institute on Drug Abuse (grant number K23DA035288) **Author conflicts of interest:** “RAB has equity ownership in Health behaviour Solutions, Inc., which is developing products for tobacco cessation that are not related to this study. The terms of this arrangement have been reviewed and approved by the University of Texas at Austin in accordance with its policy on objectivity in research. The other authors have no interests to declare.”	
***Risk of bias***		
**Bias**	**Authors' judgement**	**Support for judgement**
Random sequence generation (selection bias)	Unclear risk	Stated randomised but method not specified
Allocation concealment (selection bias)	Unclear risk	Concealment not reported
Blinding of outcome assessment (detection bias) All outcomes	Low risk	Abstinence biochemically verified
Incomplete outcome data (attrition bias) All outcomes	Low risk	At 6‐month follow‐up 21.2% (7/33) were lost to follow‐up in the intervention group and 22.2% (8/36) in the control group
Selective reporting (reporting bias)	Unclear risk	No protocol available

**Table:** Bock 2012

***Study characteristics***		
Methods	**Study design:** RCT **Location:** USA **Setting:** community **Recruitment:** advertisements in local newspapers, websites and television, flyers posted at physicians’ offices and stores **Study dates:** 2007‐10	
Participants	N = 55 **Specialist population?:** women **Definition of smoker used:** ≥ 5 cpd **Participant characteristics:** 100% female; average age: 4 years; 82% white; 35% college graduate; average cpd: 16; nicotine dependence: average FTND 5.0	
Interventions	All participants were provided with an 8‐week group CBT programme for smoking cessation, including quit day in week 2, self‐monitoring, stimulus control, coping with high‐risk situations, and stress management for smoking cessation. The programme also focused on topics of concern to women when quitting, including healthy eating, weight management, and balancing multiple roles and multiple demands. **Comparator** Group CBT + wellness classes **Mode of delivery:** face‐to‐face (group) **Intensity:** 8 CBT sessions (x 1 h) and 16 wellness classes (x 1 h) over 8 weeks **Pharmacotherapy:** none provided, but participants were permitted to use NRT or other smoking cessation medications in conjunction with the programme **Type of therapist/provider:** wellness: PhD psychologist; CBT: psychologist with > 10 years' experience in conducting smoking cessation groups **BCTs:** 1.1 Goal setting (behaviour); 1.2 Problem solving; 2.3 Self‐monitoring of behaviour; 3.1 Social support (unspecified) **Intervention:** Group CBT + Vinyasa yoga **Mode of delivery:** face‐to‐face (group) **Intensity:** 8 CBT sessions (x 1 h) and 16 yoga classes (x 1 h) over 8 weeks **Pharmacotherapy:** none provided, but participants were permitted to use NRT or other smoking cessation medications in conjunction with the programme **Type of therapist/provider** yoga: certified yoga instructors with > 15 years' experience and who were trained in the Vinyasa style CBT: psychologist with > 10 years' experience in conducting smoking cessation groups **BCTs:** 1.1 Goal setting (behaviour); 1.2 Problem solving; 2.3 Self‐monitoring of behaviour; 3.1 Social support (unspecified); 12.6 Body changes	
Outcomes	**Definition of abstinence:** 7‐day point prevalence **Longest follow‐up:** 6 months **Biochemical verification:** salivary cotinine < 15 ng/mL **Other relevant outcomes reported:** depression, anxiety, general well‐being	
Notes	**Relevant comparisons:** Vinyasa yoga + CBT vs wellness classes + CBT **Funding source:** National Center for Complementary and Alternative Medicine (AT003669) **Author conflicts of interest:** “No competing financial interests exist for any of the authors.”	
***Risk of bias***		
**Bias**	**Authors' judgement**	**Support for judgement**
Random sequence generation (selection bias)	Unclear risk	Stated randomised but method not specified
Allocation concealment (selection bias)	Unclear risk	Concealment not reported
Blinding of outcome assessment (detection bias) All outcomes	Low risk	Abstinence biochemically validated
Incomplete outcome data (attrition bias) All outcomes	Unclear risk	Attrition not reported
Selective reporting (reporting bias)	High risk	Mental health outcomes not reported in paper as specified in protocol

**Table:** Bock 2019

***Study characteristics***		
Methods	**Study design:** RCT **Location:** USA **Setting:** community **Recruitment:** advertisements on local radio stations and websites, flyers and brochures at physician’s offices and stores **Study dates:** not reported	
Participants	N = 227 **Specialist population?:** no **Definition of smoker used:** ≥ 5 cpd **Participant characteristics:** 56% female; average age: 46 years; 86% white; 28% high school education or less; average cpd: 17; nicotine dependence: average FTND 4.9	
Interventions	All participants were provided with an 8‐week group CBT programme for smoking cessation, including planning for a targeted quit day (week 4), handling smoking triggers, coping with cravings, and managing withdrawal. **Comparator** Group CBT + wellness classes **Mode of delivery:** face‐to‐face (group) **Intensity:** 8 CBT sessions (x 1 h) and 16 wellness classes (x 1 h) over 8 weeks **Pharmacotherapy:** none provided, but participants were permitted to use NRT or other smoking cessation medications in conjunction with the programme **Type of therapist/provider** smoking cessation counselling: PhD‐level psychologists wellness: wellness counsellor or other healthcare professional (e.g. sleep expert) **BCTs:** 1.1 Goal setting (behaviour), 1.2 Problem solving, 3.1 Social support, 12.6 Body changes **Intervention** Group CBT + Iyengar yoga **Mode of delivery:** face‐to‐face (group) **Intensity:** 8 CBT sessions (x 1 h) and 16 yoga classes (x 1 h) over 8 weeks **Pharmacotherapy:** none provided, but participants were permitted to use NRT or other smoking cessation medications in conjunction with the programme **Type of therapist/provider** smoking cessation counselling: PhD‐level psychologists yoga: certified Iyengar instructors with > 15 years' experience **BCTs:** 1.1 Goal setting (behaviour), 1.2 Problem solving; 3.1 Social support (unspecified)	
Outcomes	**Definition of abstinence:** continuous (based on 7‐day point prevalence at end of treatment and follow‐up) **Longest follow‐up:** 6 months **Biochemical verification:** CO < 10 ppm or salivary cotinine < 15 mg/mL **Other relevant outcomes reported:** none	
Notes	**Relevant comparisons:** Iyengar yoga + CBT vs wellness classes + CBT **Funding source:** National Institutes of Health, National Center for Complementary and Integrative Health (R01 AT006948) **Author conflicts of interest:** “The authors have no competing financial interests to declare.”	
***Risk of bias***		
**Bias**	**Authors' judgement**	**Support for judgement**
Random sequence generation (selection bias)	Low risk	Quote [protocol]: “The randomization scheme generated by the study statistician uses a permuted block randomization procedure, with small, random sized blocks. Randomization is stratified based on gender and level of nicotine dependence.”
Allocation concealment (selection bias)	Unclear risk	Concealment not reported
Blinding of outcome assessment (detection bias) All outcomes	Low risk	Abstinence biochemically verified
Incomplete outcome data (attrition bias) All outcomes	Low risk	At 6‐month follow‐up 6.2% (7/113) were lost to follow‐up in the intervention group and 4.4% (5/114) in the control group
Selective reporting (reporting bias)	Low risk	Prespecified outcomes reported

**Table:** Bricker 2014a

***Study characteristics***		
Methods	**Study design:** RCT **Location:** USA **Setting:** community **Recruitment:** uninsured callers to the South Carolina State Quitline **Study dates:** 2012‐13	
Participants	N = 121 **Specialist population?:** uninsured **Definition of smoker used:** ≥ 10 cpd for at least the past 12 months **Participant characteristics:** 69% female; average age: 39 years; 73% white; 55% high school education or less; nicotine dependence: 71% HSI score ≥ 4; 39% positive depression screen	
Interventions	**Comparator: **standard CBT‐based counselling intervention offered through the South Carolina State Quitline **Mode of delivery:** telephone **Intensity:** 5 calls (1 x 30 min, 4 x 15 min) **Pharmacotherapy:** 2 weeks of nicotine patch or gum (participant’s choice) **Type of therapist/provider:** bachelor's‐ or master's‐level providers with ≥ 3 years of general counselling experience and > 100 h of training **BCTs:** 1.2 Problem solving, 1.4 Action planning, 3.1 Social support (unspecified), 11.1: Pharmacological support, 12.4 Distraction **Intervention: **ACT programme The acceptance components taught skills in increasing willingness to experience urges that cue smoking changing the function of smoking urges responding differently to smoking urges (e.g. noticing and not acting on urges). The commitment components focused on helping individuals articulate the values guiding quitting and taking actions to quit guided by those values **Mode of delivery:** telephone **Intensity:** 5 calls (1 x 30 min, 4 x 15 min) **Pharmacotherapy:** 2 weeks of nicotine patch or gum (participant’s choice) **Type of therapist/provider:** bachelor's‐ or master's‐level providers with ≥ 3 years of general counselling experience and > 100 h of training **BCTs:** 1.4 Action planning, 3.1 Social support (unspecified), 11.1: Pharmacological support	
Outcomes	**Definition of abstinence:** 30‐day point prevalence **Longest follow‐up:** 6 months **Biochemical verification:** none **Other relevant outcomes reported:** none	
Notes	**Relevant comparisons:** ACT (telephone) + NRT vs CBT (quitline) + NRT **Funding source:** National Institute on Drug Abuse (R21DA030646) **Author conflicts of interest:** “In 2011 Dr. Heffner served as a consultant for Pfizer.”	
***Risk of bias***		
**Bias**	**Authors' judgement**	**Support for judgement**
Random sequence generation (selection bias)	Low risk	Randomised by automated algorithm
Allocation concealment (selection bias)	Low risk	Quote: “Randomized study arm assignments were computer generated and concealed from participants after eligibility was determined and consent for participation was obtained. Neither research staff nor participants had access to upcoming randomized study arm assignments”
Blinding of outcome assessment (detection bias) All outcomes	Low risk	Abstinence self‐reported but no face‐to‐face contact so no difference in intensity; differential report unlikely
Incomplete outcome data (attrition bias) All outcomes	Low risk	At 6‐month follow‐up 27.1% (16/59) were lost to follow‐up in the intervention group and 38.7% (24/62) in the control group
Selective reporting (reporting bias)	Low risk	Prespecified outcomes reported

**Table:** Bricker 2018

***Study characteristics***		
Methods	**Study design:** RCT **Location:** USA **Setting:** online **Recruitment:** targeted Facebook adverts, an online survey panel, search engine results, friends/family referral, Google ads, and earned media **Study dates:** 2014‐19	
Participants	N = 2637 **Specialist population?:** no **Definition of smoker used:** ≥ 5 cpd for the last year **Participant characteristics:** 79% female; average age: 46 years; 73% white; 28% high school education or less; nicotine dependence: average FTND 5.6; 56% current depressive symptoms; 34% current anxiety symptoms; 48% current panic disorder symptoms; 53% current PTSD symptoms; 30% current social anxiety symptoms	
Interventions	**Comparator: **Smokefree online quit programme available for 12 months **Intensity:** up to 4 daily messages (via text or email) designed to encourage logging in for 28 days after randomisation, unless they opted out **Mode of delivery:** website **Pharmacotherapy:** none **Type of therapist/provider:** N/A **BCTs:** 1.2 Problem solving, 1.4 Action planning, 5.1 Information about health consequences **Intervention: **WebQuit online ACT programme with attached online forum available for 12 months **Intensity:** up to 4 daily messages (via text or email) designed to encourage logging in for 28 days after randomisation, unless they opted out **Mode of delivery:** website **Pharmacotherapy:** none **Type of therapist/provider:** N/A **BCTs:** 1.4 Action planning, 2.3 Self‐monitoring of behaviour	
Outcomes	**Definition of abstinence:** 30‐day point prevalence **Longest follow‐up:** 12 months **Biochemical verification:** none **Other relevant outcomes reported:** none	
Notes	**Relevant comparisons:** ACT online programme (WebQuit) vs Smokefree online programme **Funding source:** National Cancer Institute (R01 CA166646; R01CA192849); National Institute on Drug Abuse (R01 DA038411) **Author conflicts of interest:** “In July 2016, Dr. Jonathan Bricker was a consultant to Glaxo Smith Kline, the makers of a nicotine replacement therapy. He now serves on the Scientific Advisory Board of Chrono Therapeutics, the makers of a nicotine replacement therapy device. Other authors have no declarations.”	
***Risk of bias***		
**Bias**	**Authors' judgement**	**Support for judgement**
Random sequence generation (selection bias)	Low risk	Quote: “Using randomly permuted block randomization, stratified by daily smoking frequency (≤20 vs. ≥21), education (≤ high school vs. ≥ some college), and gender (male vs. female).”
Allocation concealment (selection bias)	Low risk	Quote: “Random assignments were concealed from participants until after study eligibility, consent, and baseline data was obtained. Neither research staff nor study participants had access to upcoming randomized study arm assignments.”
Blinding of outcome assessment (detection bias) All outcomes	Low risk	Abstinence self‐reported but no face‐to‐face contact so no difference in intensity; differential report unlikely
Incomplete outcome data (attrition bias) All outcomes	Low risk	At 12‐month follow‐up 13.5% (178/1319) were lost to follow‐up in the intervention group and 11.4% (150/1318) in the control group
Selective reporting (reporting bias)	Low risk	Prespecified outcomes reported

**Table:** Bricker 2020

***Study characteristics***		
Methods	**Study design:** RCT **Location:** USA **Setting:** online **Recruitment:** nationally via Facebook ads, a survey sampling company, search engine results, and referral from friends and family **Study dates:** 2017‐19	
Participants	N = 2415 **Specialist population?:** no **Definition of smoker used:** ≥ 5 cpd for at least the past year **Participant characteristics:** 70% female; average age: 38 years; 69% white; 41% high school education or less; nicotine dependence: average FTND 5.9; 48.5% positive depression screen	
Interventions	**Comparator: **QuitGuide smartphone app including content on motivations to quit, preparing to quit (including quit plan, triggers, social support, and advice on pharmacotherapy), avoiding cravings, and remaining abstinent **Mode of delivery:** smartphone app **Pharmacotherapy:** none **BCTs:** 1.1 Goal setting (behaviour), 1.2 Problem solving, 3.1 Social support (unspecified), 5.1 Information about health consequences, 8.2 Behaviour substitution **Intervention: **iCanQuit smartphone app; ACT programme teaching skills for coping with urges, staying motivated and preventing relapse, and including advice on pharmacotherapy **Mode of delivery:** smartphone app **Pharmacotherapy:** none **BCTs:** 1.1 Goal setting (behaviour), 1.2 Problem solving, 2.3 Self‐monitoring of behaviour, 4.1 Instruction on how to perform behaviour (where to and what NRTs)	
Outcomes	**Definition of abstinence:** prolonged **Longest follow‐up:** 12 months **Biochemical verification:** none **Other relevant outcomes reported:** none	
Notes	**Relevant comparisons:** ACT app (iCanQuit) vs QuitGuide app **Funding source:** National Cancer Institute (R01CA192849) **Author conflicts of interest:** “Dr Bricker reported receiving grants from the National Cancer Institute during the conduct of the study; serving on the scientific advisory board for and receiving personal fees from Chrono Therapeutics outside the submitted work; and reported that the Fred Hutchinson Cancer Research Center has applied for a US patent that pertains to the content of the iCanQuit application. 2Morrow, Inc, a Kirkland, Washington–based software company, has licensed this technology from the Fred Hutchinson Cancer Research Center. Dr Bricker had no personal financial relationships with this patent application, the licensing agreement, or 2Morrow, Inc. Ms Mull reported receiving grants from the National Institutes of Health/National Cancer Institute during the conduct of the study. Dr Heffner reported receiving nonfinancial support from Pfizer outside the submitted work. None of the authors has a financial relationship with the iCanQuit application and thus will not receive any compensation when it becomes publicly available. No other disclosures were reported.”	
***Risk of bias***		
**Bias**	**Authors' judgement**	**Support for judgement**
Random sequence generation (selection bias)	Low risk	Quote: “The random allocation sequence was generated by a database manager and implemented automatically by the study website.”
Allocation concealment (selection bias)	Low risk	Quote: “Random assignments were concealed from participants throughout the trial. The random allocation sequence was generated by a database manager and implemented automatically by the study website. Neither research staff nor study participants had access to upcoming randomized study group assignments.”
Blinding of outcome assessment (detection bias) All outcomes	Low risk	Abstinence self‐reported but no face‐to‐face contact so no difference in intensity; differential report unlikely
Incomplete outcome data (attrition bias) All outcomes	Low risk	At 12‐month follow‐up 30.9% (375/1214) were lost to follow‐up in the intervention group and 27.5% (330/1201) in the control group
Selective reporting (reporting bias)	Low risk	Prespecified outcomes reported

**Table:** Brown 2013

***Study characteristics***		
Methods	**Study design:** RCT **Location:** USA **Setting:** unclear, recruitment from community **Recruitment:** newspaper and radio advertisements targeting smokers who had “previous difficulty quitting for even short periods of time” **Study dates:** not reported	
Participants	N = 49 **Specialist population?:** smokers with a history of early lapse (< 72 h post‐quit) **Definition of smoker used:** ≥ 15 cpd for at least the past 3 years **Participant characteristics:** 49% female; average age: 48 years; 90% white; 33% high school education or less; average cpd: 22; nicotine dependence: average FTND 6.3; 29% one or more depressive episodes	
Interventions	**Comparator: **standard treatment covering self‐monitoring, identifying triggers, developing self‐management strategies for coping with triggers, and relapse prevention skills **Mode of delivery:** face‐to‐face (group), telephone **Intensity:** 6 face‐to‐face group sessions (x 90 min) and 1 phone call (20 min) over 6 weeks **Pharmacotherapy:** 8 weeks of nicotine patches from quit date (4 weeks x 21 mg, 2 x 14 mg, 2 x 7 mg) **Type of therapist/provider:** doctoral‐level psychologists or trainees (trained by senior investigators) **BCTs:** 1.2 Problem solving, 2.3 Self‐monitoring of behaviour, 8.2 Behaviour substitution; 11.1 Pharmacological support **Intervention: **distress tolerance treatment. This included exercises aimed at increasing participants’ tolerance of distress while maintaining a focus on the valued life goals associated with quitting smoking **Mode of delivery:** face‐to‐face (individual and group) **Intensity:** 6 individual sessions (x 50 min) and 9 group sessions (x 2 h) over 8 weeks **Pharmacotherapy:** 8 weeks of nicotine patches from quit date (4 weeks x 21 mg, 2 x 14 mg, 2 x 7 mg) **Type of therapist/provider:** doctoral‐level psychologists or trainees (trained by senior investigators) **BCTs:** 7.3 Reduce prompts/cues: 'nicotine fading', 11.1 pharmacological support	
Outcomes	**Definition of abstinence:** 7‐day point prevalence **Longest follow‐up:** 6 months **Biochemical verification:** CO ≤ 5 ppm and cotinine ≤ 10 ng/mL **Other relevant outcomes reported:** negative affect	
Notes	**Relevant comparisons:** distress tolerance training + NRT vs standard treatment + NRT **Funding source:** National Institute on Drug Abuse (DA017332) **Author conflicts of interest:** “None declared”	
***Risk of bias***		
**Bias**	**Authors' judgement**	**Support for judgement**
Random sequence generation (selection bias)	Unclear risk	Method not reported
Allocation concealment (selection bias)	Unclear risk	Concealment not reported
Blinding of outcome assessment (detection bias) All outcomes	Low risk	Abstinence biochemically verified
Incomplete outcome data (attrition bias) All outcomes	Low risk	At 6‐month follow‐up 7.4% (2/27) were lost to follow‐up in the intervention group and 4.8% (1/21) in the control group
Selective reporting (reporting bias)	Unclear risk	No protocol available

**Table:** Davis 2014a

***Study characteristics***		
Methods	**Study design:** RCT **Location:** USA **Setting:** community **Recruitment:** advertisements on television, newspaper, and flyers, with ad placement in low‐SES neighbourhoods **Study dates:** 2010‐13	
Participants	N = 196 **Specialist population?:** moderately low SES **Definition of smoker used:** ≥ 5 cpd **Participant characteristics:** 50% female; average age: 42 years; 77% white; 49% high school education or less; average cpd: 16; average number of years smoked: 22	
Interventions	**Comparator: **smoking cessation counselling through the Wisconsin Tobacco Quit Line **Mode of delivery:** telephone **Intensity:** encouraged to make repeated calls to the Quit Line, one visit to study centre to collect nicotine patches **Pharmacotherapy:** 4 weeks of nicotine patches (21 mg) from quit date **Type of therapist/provider:** not reported **BCTs:** 1.1 Goal setting (behaviour), 11.1 Pharmacological support **Intervention: **Mindfulness Training for Smokers (MTS) **Mode of delivery:** face‐to‐face (group), video **Intensity:** 10 sessions (total of 32 h) over 8 weeks **Pharmacotherapy:** 4 weeks of nicotine patches (21 mg) from quit date **Type of therapist/provider:** instructors with no formal addiction training, but with successful completion of the 2‐day MTS Instructor Training Course **BCTs:** 1.2 Problem solving, 3.1 Social support, 4.1 Instruction on how to perform behaviour, 11.1 Pharmacological support, 11.2 Reducing negative emotions, 12.6 Body changes	
Outcomes	**Definition of abstinence:** continuous **Longest follow‐up:** 6 months **Biochemical verification:** CO < 7 ppm **Other relevant outcomes reported:** none	
Notes	**Relevant comparisons:** mindfulness training + NRT vs quit line counselling + NRT **Funding source:** National Institute on Drug Abuse (K23DA022471) **Author conflicts of interest:** Not reported	
***Risk of bias***		
**Bias**	**Authors' judgement**	**Support for judgement**
Random sequence generation (selection bias)	Unclear risk	Insufficient detail to make a judgment Quote: “Consented individuals were asked to complete baseline assessment visit (carbon monoxide (CO) breath testing and self‐report measures) and then undergo randomization via random draws to either the Control Group (Quit Line + 4 weeks Nicotine Replacement Therapy (NRT)) or MTS (MTS + 4 weeks NRT)”
Allocation concealment (selection bias)	Unclear risk	Concealment not reported
Blinding of outcome assessment (detection bias) All outcomes	Low risk	Abstinence biochemically verified
Incomplete outcome data (attrition bias) All outcomes	High risk	Attrition was high in both groups: 78.1% (82/105) in the intervention group and 64.8% (59/91) in the control group
Selective reporting (reporting bias)	Low risk	Prespecified abstinence outcomes were reported. However, the depression, anxiety and stress outcome (DASS) was not reported for both arms

**Table:** Davis 2014b

***Study characteristics***		
Methods	**Study design:** RCT **Location:** USA **Setting:** community **Recruitment:** advertisements placed on television, newspaper, and flyers (targeted in low‐SES areas) **Study dates:** 2011‐12	
Participants	N = 135 **Specialist population?:** people living in low‐SES areas **Definition of smoker used:** ≥ 5 cpd, uses no other tobacco products **Participant characteristics:** 47% female; average age: 45 years; 88% white; 35% high school education or less; average cpd: 18; nicotine dependence: average FTND 4.8	
Interventions	**Comparator: **American Lung Association's Freedom from Smoking (FFS) programme, enhanced to match the Mindfulness Training for Smokers (MTS) intervention more closely (FFS‐E) **Mode of delivery:** face‐to‐face (group), video, audio recording **Intensity:** 24 h over 7 weeks **Pharmacotherapy:** 2 weeks of nicotine patches from quit date **Type of therapist/provider:** master's degree in psychology, no specialised training or certification in addiction therapy (FFS instructors are typically laypeople), provided with a 2‐day FFS‐E teacher‐training course **BCTs:** 1.1 Goal setting (behaviour): set a quit plan, 11.1 Pharmacological support, 12.6 Body changes **Intervention: **Mindfulness Training for Smokers (MTS) **Mode of delivery:** face‐to‐face (group), video, audio recording **Intensity:** 7 classes (x 2.5 h) and a quit day retreat (6.5 h) over 7 weeks (total 24 h) **Pharmacotherapy:** 2 weeks of nicotine patches from quit date **Type of therapist/provider:** master's degree in psychology (except for one who had a PhD in Sociology), no specialised training or certification in addiction therapy, provided with a 2‐day MTS teacher‐training course **BCTs:** 1.2 Problem solving, 3.1 Social support (unspecified), 4.1 Instruction on how to perform behaviour, 11.1 Pharmacological support, 11.2 Reducing negative emotions, 12.6 Body changes	
Outcomes	**Definition of abstinence:** 7‐day point prevalence **Longest follow‐up:** 6 months **Biochemical verification:** CO < 7 ppm **Other relevant outcomes reported:** stress	
Notes	**Relevant comparisons:** mindfulness training + NRT vs Freedom From Smoking programme + NRT **Funding source:** National Institute on Drug Abuse (K23 DA022471) **Author conflicts of interest:** not reported	
***Risk of bias***		
**Bias**	**Authors' judgement**	**Support for judgement**
Random sequence generation (selection bias)	Unclear risk	Method not reported
Allocation concealment (selection bias)	Unclear risk	Concealment not reported
Blinding of outcome assessment (detection bias) All outcomes	Low risk	Abstinence biochemically verified
Incomplete outcome data (attrition bias) All outcomes	High risk	Attrition was high in both groups: 57.4% (39/68) in the intervention group and 55.2% (37/67) in the control group
Selective reporting (reporting bias)	Low risk	Prespecified outcomes reported

**Table:** de Souza 2020

***Study characteristics***		
Methods	**Study design:** RCT **Location:** Brazil **Setting:** outpatient public health tobacco treatment service **Recruitment:** by phone from a waiting list of an outpatient public health tobacco treatment service **Study dates:** 2012‐16	
Participants	N = 86 **Specialist population?:** no **Definition of smoker used:** ≥ 10 cpd **Participant characteristics:** 80% female; average age: 50 years; 85% high school education or less; 33.7% average cpd ≥ 20	
Interventions	**Comparator: **standard treatment (CBT + maintenance sessions) **Mode of delivery:** unclear **Intensity:** smoking cessation phase: 4 weekly CBT sessions (x 90 min; smoking cessation phase) maintenance phase: 6 CBT sessions between weeks 6 and 48 **Pharmacotherapy:** choice of NRT or bupropion **Type of therapist/provider:** physician with experience treating smoking and with training in the standard treatment approach **BCTs:** 5.1 Information about health consequences, 11.1 Pharmacological support **Intervention: **standard treatment (CBT + maintenance sessions) + mindfulness‐based relapse prevention (MBRP) **Mode of delivery:** group sessions, audio CD **Intensity:** smoking cessation phase: 4 weekly CBT sessions (x 90 min; smoking cessation phase) maintenance phase: 6 CBT sessions between weeks 6 and 48 + 8 weekly group MBRP sessions (x 2 h) **Pharmacotherapy:** choice of NRT or bupropion **Type of therapist/provider:** certified MBRP instructor who had received MBRP training; physician with experience treating smoking and with training in the standard treatment approach **BCTs:** 1.2 Problem solving, 2.3 Self‐monitoring (behaviour), 5.1 Information about health consequences, 11.1 Pharmacological support, 12.6 Body changes	
Outcomes	**Definition of abstinence:** CO < 10 ppm **Longest follow‐up:** 6 months **Biochemical verification:** CO < 10 ppm **Other relevant outcomes reported:** depression, anxiety, positive and negative affect	
Notes	**Relevant comparisons:** mindfulness‐based relapse prevention + CBT + NRT/bupropion vs CBT + NRT/bupropion **Funding source:** Fundação de Amparo à Pesquisa do Estado de São Paulo (2013/02316‐5), Conselho Nacional de Desenvolvimento Científico e Tecnológico (870470/1997‐3), Fundação de Amparo à Pesquisa do Estado de Minas Gerais (APQ‐04279‐10) and Coordenação de Aperfeiçoamento de Pessoal de Nível Superior (552452/2011) **Author conflicts of interest:** “None declared.”	
***Risk of bias***		
**Bias**	**Authors' judgement**	**Support for judgement**
Random sequence generation (selection bias)	Unclear risk	Method not reported
Allocation concealment (selection bias)	Unclear risk	Concealment not reported
Blinding of outcome assessment (detection bias) All outcomes	Low risk	Abstinence biochemically verified
Incomplete outcome data (attrition bias) All outcomes	High risk	Attrition was high in both groups: at 24‐week follow‐up 75.0% (33/44) were lost to follow‐up in the intervention group and 78.6% (33/42) in the control group
Selective reporting (reporting bias)	High risk	Some prespecified outcomes not reported (dependence, maintenance of abstinence at 12 months, change in smoking urges)

**Table:** Garrison 2020

***Study characteristics***		
Methods	**Study design:** RCT **Location:** USA **Setting:** online **Recruitment:** online ads on Google, Reddit and smokefree.gov website, social media posts, word of mouth, and blog posts **Study dates:** 2014‐16	
Participants	N = 325 **Specialist population?:** no **Definition of smoker used:** ≥ 5 cpd, ≤ 3 months past‐year abstinence **Participant characteristics:** 72% female; average age: 41 years; 81% white; 16% high school education or less; average cpd: 16	
Interventions	**Comparator: **experience sampling (ES) only **Mode of delivery:** smartphone app **Intensity:** 22 days of check‐ins 6 x a day **Pharmacotherapy:** none **BCTs:** 2.3 Self‐monitoring of behaviour **Intervention: **mobile mindfulness training with experience sampling (MMT‐ES) **Mode of delivery:** smartphone app **Intensity:** 22 days of training modules (5–15 min/d) **Pharmacotherapy:** none **BCTs:** 2.3 Self‐monitoring of behaviour, 2.4 Self‐monitoring of outcome(s) of behaviour, 12.6 Body changes: body scan	
Outcomes	**Definition of abstinence:** 7‐day point prevalence **Longest follow‐up:** 6 months **Biochemical verification:** CO < 10 ppm verified by video by a blinded researcher **Other relevant outcomes reported:** none	
Notes	**Relevant comparisons:** mobile mindfulness training app + experience sampling vs experience sampling **Funding source:** American Heart Association [14CRP18200010] and National Institute on Drug Abuse grant [K12DA00167] **Author conflicts of interest:** “Judson A. Brewer and Prasanta Pal own stock in Claritas Mindsciences, the company that developed the apps used in this study. All other authors declare that they have no competing interests.”	
***Risk of bias***		
**Bias**	**Authors' judgement**	**Support for judgement**
Random sequence generation (selection bias)	Unclear risk	Method not reported
Allocation concealment (selection bias)	Unclear risk	Concealment not reported
Blinding of outcome assessment (detection bias) All outcomes	Low risk	Abstinence biochemically verified
Incomplete outcome data (attrition bias) All outcomes	Low risk	At 6‐month follow‐up 21.7% (31/143) were lost to follow‐up in the intervention group and 25.4% (47/185) in the control group
Selective reporting (reporting bias)	Low risk	Prespecified abstinence outcomes were reported. However, 4‐item Perceived Stress Scale (PSS) was not reported

**Table:** Gaskins 2015

***Study characteristics***		
Methods	**Study design:** RCT **Location:** USA **Setting:** community **Recruitment:** advertisements on radio and local newspapers, flyers in local papers and posted at local venues (e.g. pharmacies, supermarkets), and brief descriptions of the study listed in guides published by local yoga studios **Study dates:** 2009‐11	
Participants	N = 38 **Specialist population?:** men **Definition of smoker used:** ≥ 5 cpd for the past year **Participant characteristics:** 0% female; average age: 40 years; 95% white; 11% less than high school education; 13% household income < USD 10,000; average cpd: 19; nicotine dependence: average FTND 4.8	
Interventions	**Comparator: **CBT + wellness classes **Mode of delivery:** face‐to‐face (individual), written materials, video **Intensity:** 8 CBT sessions (x 30 min) each followed by a brief wellness discussion over 8 weeks **Pharmacotherapy:** none **Type of therapist/provider:** CBT: doctoral‐level counsellor; wellness: smoking counsellor **BCTs:** 1.1 Goal setting (behaviour), 1.2 Problem solving, 2.3 Self monitoring of behaviour, 3.1 Social support, 11.2 Reduce negative emotions **Intervention:** CBT + Vinyasa yoga **Mode of delivery:** face‐to‐face (individual CBT, group yoga) **Intensity:** 16 Vinyasa yoga classes (x 60–90 min) and 8 CBT sessions (x 30 min) over 8 weeks **Pharmacotherapy:** none **Type of therapist/provider:** CBT: doctoral‐level counsellor; yoga: certified yoga instructors **BCTs:** 1.1 Goal setting (behaviour), 1.2 Problem solving, 2.3 Self‐monitoring of behaviour, 3.1 Social support (unspecified), 11.2 Reduce negative emotions, 12.6 Body changes (yoga and relaxation)	
Outcomes	**Definition of abstinence:** 7‐day point prevalence **Longest follow‐up:** 6 months **Biochemical verification:** CO < 8 ppm **Other relevant outcomes reported:** depression, anxiety	
Notes	**Relevant comparisons:** Vinyasa yoga + CBT vs wellness classes + CBT **Funding source:** National Institutes of Health, National Center for Complementary, and Alternative Medicine (R21AT003669) **Author conflicts of interest:** “Ronnesia B. Gaskins, Ernestine Jennings, Herpreet Thind, Joseph Fava, Santina Horowitz, Ryan Lantini, Bruce M. Becker, and Beth C. Bock declare that they have no conflict of interest.”	
***Risk of bias***		
**Bias**	**Authors' judgement**	**Support for judgement**
Random sequence generation (selection bias)	Unclear risk	Method not reported
Allocation concealment (selection bias)	Unclear risk	Concealment not reported
Blinding of outcome assessment (detection bias) All outcomes	Low risk	Abstinence biochemically verified
Incomplete outcome data (attrition bias) All outcomes	High risk	Attrition was different across groups, with high attrition in the yoga group: 56.5% (13/23) in the intervention group vs. 26.7% (4/15) in the control group
Selective reporting (reporting bias)	Low risk	Prespecified outcomes reported

**Table:** Gifford 2003

***Study characteristics***		
Methods	**Study design:** RCT **Location:** USA **Setting:** community **Recruitment:** through newspaper and radio advertisements and flyers **Study dates:** not reported	
Participants	N = 102 **Specialist population?:** no **Definition of smoker used:** self‐identified nicotine dependence, ≥ 10 cpd for the last 12 months **Participant characteristics:** 59% female; average age: 43 years; 77% white; 4% some high school or less; average cpd: 21; nicotine dependence: average FTND 5.4	
Interventions	**Comparator: **nicotine replacement therapy **Mode of delivery:** face‐to‐face **Intensity:** 1‐h session plus weekly return visits to receive new patches for following week **Pharmacotherapy:** 7 weeks of nicotine patches (22 mg/d for 4 weeks, followed by patches of 11 mg/d for 3 weeks) **Type of therapist/provider:** psychiatrist with extensive training in the medical management of smoking cessation, including NRT, or psychiatry resident under supervision of psychiatrist **BCTs:** 4.1 Instruction on how to perform behaviour, 11.1 Pharmacological support **Intervention:** ACT **Mode of delivery:** face‐to‐face (individual and group), telephone call if missed face‐to‐face individual session **Intensity:** 7 individual sessions and 7 group sessions over 7 weeks (duration of sessions not reported) **Pharmacotherapy:** none **Type of therapist/provider:** ACT therapist **BCTs:** 1.1 Goal setting (behaviour), 1.2 Problem solving, 1.9: Commitment, 7.3 Reduce prompts and cues, 8.7 Graded tasks, 12.6 Body changes	
Outcomes	**Definition of abstinence:** 7‐day point prevalence **Longest follow‐up:** 12 months **Biochemical verification:** CO < 11 ppm **Other relevant outcomes reported:** negative affect	
Notes	**Relevant comparisons:** ACT vs NRT **Funding source:** National Institutes of Health, National Cancer Institute (CA84813) **Author conflicts of interest:** not reported	
***Risk of bias***		
**Bias**	**Authors' judgement**	**Support for judgement**
Random sequence generation (selection bias)	Low risk	Quote: “If participants were accepted into the study, they were randomly assigned to treatment condition using a random numbers generator”
Allocation concealment (selection bias)	Unclear risk	Concealment not reported
Blinding of outcome assessment (detection bias) All outcomes	Low risk	Abstinence biochemically verified
Incomplete outcome data (attrition bias) All outcomes	Low risk	At 12‐month follow‐up 38.3% (18/47) were lost to follow‐up in the intervention group and 23.6% (13/55) in the control group
Selective reporting (reporting bias)	Unclear risk	No protocol available

**Table:** Mak 2020

***Study characteristics***		
Methods	**Study design:** RCT **Location:** Hong Kong **Setting:** primary care **Recruitment:** from 6 primary healthcare centres **Study dates:** 2012‐15	
Participants	N = 144 **Specialist population?:** no **Definition of smoker used:** ≥ 1 cpd in the past 30 days **Participant characteristics:** 29% female; average age: 46 years; 22% primary education or less; 18% unemployed; 16% household income ≤ HKD 9999; 17% average cpd > 20; 38% high nicotine dependence	
Interventions	**Comparator: **brief advice + self‐help materials **Mode of delivery:** face‐to‐face, written materials **Intensity:** brief 5 minute talk **Pharmacotherapy:** none **Type of therapist/provider:** not reported **BCTs:** 1.2 Problem solving, 4.1 Instruction on how to perform the behaviour, 5.1 Information about health consequences **Intervention: **ACT + self‐help materials **Mode of delivery:** face‐to‐face, telephone, written materials **Intensity:** 1 face‐to‐face ACT session and 2 telephone follow‐up sessions (each 15‐20 min) **Pharmacotherapy:** none **Type of therapist/provider:** experienced health counsellor trained in the principles of ACT applied in smoking cessation **BCTs:** 1.2 Problem solving, 4.1 instruction on how to perform the behaviour, 5.3 Information about social and environmental consequences, 12.6 Body changes	
Outcomes	**Definition of abstinence:** 7‐day point prevalence **Longest follow‐up:** 12 months **Biochemical verification:** CO < 6 ppm was measured but validated quit rates are not reported **Other relevant outcomes reported:** none	
Notes	**Relevant comparisons:** ACT + self‐help vs brief advice + self‐help **Funding source:** Health and Medical Research Fund of the Food and Health Bureau of the Hong Kong SAR Government (10111861) **Author conflicts of interest:** “The authors declare that they have no competing interests.”	
***Risk of bias***		
**Bias**	**Authors' judgement**	**Support for judgement**
Random sequence generation (selection bias)	Low risk	Quote: “The process of randomization was based on computer‐generated, block randomization with random block sizes, which were placed in sealed opaque envelopes."
Allocation concealment (selection bias)	Low risk	Quote: “The process of randomization was based on computer‐generated, block randomization with random block sizes, which were placed in sealed opaque envelopes."
Blinding of outcome assessment (detection bias) All outcomes	High risk	Although abstinence was biochemically verified, only unverified quit rates are reported and we were unable to obtain verified rates from the study authors.
Incomplete outcome data (attrition bias) All outcomes	High risk	Attrition was high: at 12‐month follow‐up 50.0% (35/70) were lost to follow‐up in the intervention group and 58.1% (43/74) in the control group
Selective reporting (reporting bias)	Unclear risk	Abstinence was defined as prespecified, although 12 month follow‐up was not prespecified. 6 month rates are not reported so unclear whether the reporting of 12‐month rates is an example of selective reporting

**Table:** McClure 2020

***Study characteristics***		
Methods	**Study design:** RCT **Location:** USA **Setting:** healthcare clinics **Recruitment:** potential participants were identified via automated medical records **Study dates:** 2011‐15	
Participants	N = 450 **Specialist population?:** no **Definition of smoker used:** ≥ 10 cpd **Participant characteristics:** 53% female; average age: 51 years; 83% white; 22% high school education or less; nicotine dependence: average FTND 4.9; 30% current depression; 12% current anxiety	
Interventions	**Comparator: **CBT, including motivational content (the risks of smoking and benefits of quitting, understanding one’s personal reasons for quitting), behavioural exercises (tracking one’s smoking), and psychoeducational content (how to use the nicotine patch, how to set a quit date) **Mode of delivery:** face‐to‐face (group) **Intensity:** 5 sessions (x 90 min) over 5 weeks **Pharmacotherapy:** 8 weeks of nicotine patches, adjusted to individual cpd **Type of therapist/provider:** master’s‐level counsellor with training in CBT, supervised by licensed clinical psychologists with expertise in CBT **BCTs:** 1.1 Goal setting (behaviour), 1.2 Problem solving, 1.4 Action planning (action plan review), 1.9 Commitment, 3.1 Social support (unspecified), 11.1 Pharmacological support, 12.6 Body changes **Intervention: **ACT **Mode of delivery:** face‐to‐face (group) **Intensity:** 5 sessions (x 90 min) over 5 weeks **Pharmacotherapy:** 8 weeks of nicotine patches, adjusted to individual cpd **Type of therapist/provider:** master’s‐level counsellor with training in ACT, supervised by licensed clinical psychologists with expertise in ACT **BCTs:** 1.1 Goal setting (behaviour), 1.2 Problem solving, 1.4 Action planning (action plan review), 1.9 Commitment, 3.1 Social support (unspecified), 11.1 Pharmacological support, 12.6 Body changes	
Outcomes	**Definition of abstinence:** 30‐day point prevalence **Longest follow‐up:** 12 months **Biochemical verification:** salivary cotinine < 15 ng/mL **Other relevant outcomes reported:** none	
Notes	**Relevant comparisons:** ACT + NRT vs CBT + NRT **Funding source:** National Cancer Institute (R01CA151251) **Author conflicts of interest:** “In July 2016, JB was a consultant to GlaxoSmithKline, the makers of a nicotine‐replacement therapy. He now serves on the Scientific Advisory Board of Chrono Therapeutics, the makers of a nicotine‐replacement therapy device. JLH has also received support from Pfizer. No other authors have conflicts to disclose.”	
***Risk of bias***		
**Bias**	**Authors' judgement**	**Support for judgement**
Random sequence generation (selection bias)	Unclear risk	Method not reported
Allocation concealment (selection bias)	Unclear risk	Concealment not reported
Blinding of outcome assessment (detection bias) All outcomes	Low risk	Abstinence biochemically verified
Incomplete outcome data (attrition bias) All outcomes	Low risk	At 12‐month follow‐up 22.8% (51/224) were lost to follow‐up in the intervention group and 22.1% (50/226) in the control group
Selective reporting (reporting bias)	Low risk	Prespecified outcomes reported

**Table:** O'Connor 2020

***Study characteristics***		
Methods	**Study design:** RCT **Location:** Ireland **Setting:** community **Recruitment:** from the community by self‐selection **Study dates:** 2016‐18	
Participants	N = 150 **Specialist population?:** no **Definition of smoker used:** ≥ 10 cpd for the past 12 months **Participant characteristics:** 53% female; average age: 36 years; average years in education: 16.7; 75% employed; average cpd: 17; nicotine dependence: average FTND 4.7	
Interventions	**Comparator: **behavioural support programme based on motivational interviewing **Mode of delivery:** face‐to‐face (group) **Intensity:** 6 sessions (x 90 min) over 6 weeks **Pharmacotherapy:** none **Type of therapist/provider:** a doctoral‐level and a bachelor’s‐level staff member who had undergone training in smoking cessation from the National Centre for Smoking Cessation and Training (NCSCT) **BCTs:** 1.2 Problem solving, 1.4 Action planning, 5.1 Information about health consequences **Intervention 1: **ACT **Mode of delivery:** face‐to‐face (group) **Intensity:** 6 sessions (x 90 min) over 6 weeks **Pharmacotherapy:** none **Type of therapist/provider:** psychology doctoral student with training in ACT and a doctoral‐level peer‐reviewed ACT trainer **BCTs:** 1.4 Action planning, 12.6 Body changes **Intervention 2: **ACT + SmartQuit smartphone app **Mode of delivery:** face‐to‐face (group), smartphone app **Intensity:** 6 sessions (x 90 min) over 6 weeks **Pharmacotherapy:** none **Type of therapist/provider:** psychology doctoral student with training in ACT and a doctoral‐level peer‐reviewed ACT trainer **BCTs:** 1.2 Problem solving, 1.4 Action planning, 12.6 Body changes	
Outcomes	**Definition of abstinence:** 7‐day point prevalence **Longest follow‐up:** 6 months **Biochemical verification:** CO < 10 ppm **Other relevant outcomes reported:** positive mental health	
Notes	**Relevant comparisons:** ACT (face‐to‐face + app) vs ACT (face‐to‐face) ACT (face‐to‐face) vs behavioural support **Funding source:** Irish Research Council Government of Ireland Postgraduate Scholarship **Author conflicts of interest:** “The authors declare that there are no conflicts of interest.”	
***Risk of bias***		
**Bias**	**Authors' judgement**	**Support for judgement**
Random sequence generation (selection bias)	Low risk	Quote: “the allocation sequence was generated with random block sizes of 3, 6 and 9 by a researcher with no clinical involvement in the trial using an online randomization tool”
Allocation concealment (selection bias)	Low risk	Quote: “allocation sequence was concealed from the researcher (MOC) enrolling participants in sequentially numbered, opaque, sealed envelopes”
Blinding of outcome assessment (detection bias) All outcomes	Low risk	Abstinence biochemically verified
Incomplete outcome data (attrition bias) All outcomes	Low risk	At 6‐month follow‐up 12.0% (6/50) were lost to follow‐up in the combined ACT + app group, 6.0% (3/50) in the ACT group, and 18.0% (9/50) in the control group
Selective reporting (reporting bias)	Low risk	Prespecified outcomes reported

**Table:** Pbert 2020

***Study characteristics***		
Methods	**Study design:** cluster‐RCT **Location:** USA **Setting:** high schools **Recruitment:** parents of all 9‐12 grade students were sent letters allowing them to opt out of their adolescent participating in the study **Study dates:** 2014‐16	
Participants	N = 146 (across 9 schools) **Specialist population?:** high school students **Definition of smoker used:** average of ≥ 5 cpd for the past 7 days **Participant characteristics:** 56% female; average age: 17 years; 90% white; 69% in reduced or free lunch programme; average cpd: 5; 23% high level of nicotine dependence; 43% elevated depressive symptoms	
Interventions	All participants had weekly visits with the school nurse for 4 weeks, plus written self‐help materials or 1 of 2 smartphone apps **Comparator 1: **written self‐help materials (pamphlets) **Mode of delivery:** written materials, face‐to‐face **Intensity:** weekly visits with the school nurse over 4 weeks **Pharmacotherapy:** none **Type of therapist/provider:** school nurse **BCTs:** 1.2 Problem solving, 4.1 Instruction on how to perform behaviour **Comparator 2: **National Cancer Institute QuitSTART App, a free smartphone app developed as a smoking cessation resource for teens **Mode of delivery:** smartphone app, face‐to‐face **Intensity:** app intensity unclear, weekly visits with the school nurse over 4 weeks **Pharmacotherapy:** none **Type of therapist/provider:** school nurse, smartphone app **BCTs:** 1.1 Goal setting (behaviour), 1.2 Problem solving, 2.3 Self‐monitoring of behaviour, 15.4 Self‐talk **Intervention: **craving to quit (C2Q) app adapted for teens, based on core elements of mindfulness training for smoking **Mode of delivery:** smartphone app, face‐to‐face **Intensity:** 22 training modules plus 4 bonus modules (5‐15 min/module), weekly visits with the school nurse over 4 weeks **Pharmacotherapy:** none **Type of therapist/provider:** school nurse, smartphone app **BCTs:** 1.1 Goal setting (behaviour), 1.2 Problem solving, 2.3 Self‐monitoring of behaviour	
Outcomes	**Definition of abstinence:** 7‐day point prevalence **Longest follow‐up:** 6 months **Biochemical verification:** salivary cotinine < 11.4 ng/mL **Other relevant outcomes reported:** none	
Notes	**Relevant comparisons:** mindfulness training app (C2Q) + school nurse vs QuitSTART app + school nurse mindfulness training app (C2Q) + school nurse vs self‐help materials + school nurse **Funding source:** National Institute on Drug Abuse (R34 DA037886) **Author conflicts of interest:** "JB owns stock in Claritas MindSciences, the company that developed the Craving to Quit app. The other authors declare that they have no competing interest."	
***Risk of bias***		
**Bias**	**Authors' judgement**	**Support for judgement**
Random sequence generation (selection bias)	Unclear risk	Method not reported
Allocation concealment (selection bias)	Unclear risk	Concealment not reported
Blinding of outcome assessment (detection bias) All outcomes	Low risk	Abstinence biochemically verified
Incomplete outcome data (attrition bias) All outcomes	Low risk	At 6‐month follow‐up 8.3% (4/48) were lost to follow‐up in the intervention (C2Q) group, 2.0% (1/50) in the QuitSTART group, and 4.2% (2/48) in the materials group
Selective reporting (reporting bias)	Low risk	Prespecified outcomes reported

**Table:** Savvides 2014

***Study characteristics***		
Methods	**Study design:** RCT **Location:** Cyprus **Setting:** high schools and universities **Recruitment:** from the cafeterias and psychology classes of the universities and through the Ministry of Education and head teachers of the schools **Study dates:** not reported	
Participants	N = 165 **Specialist population?:** young adults **Definition of smoker used:** ≥ 1 cpd **Participant characteristics:** 65% female; average age: 22 years; 32% weekly allowance/income < EUR 50; average cpd: 9; average FTND score: 3.1	
Interventions	**Comparator: **waitlist **Intensity:** none **Pharmacotherapy:** none **Intervention: **avatar‐led, internet‐based, ACT **Mode of delivery:** online **Intensity:** 6 sessions (x 25 min) spaced out over a minimum of 3 days between each session **Pharmacotherapy:** none **BCTs:** 1.1 Goal setting (behaviour), 1.2 Problem solving, 1.5 Review behavioural goals, 1.9 Commitment, 10.3 Non‐specific reward, 11.2 Reducing negative emotions, 12.3 Avoidance/Reducing exposure to cues for the behaviour, 12.6 Body changes, 13.4 Valued self‐identity	
Outcomes	**Definition of abstinence:** 30‐day point prevalence **Longest follow‐up:** 12 months **Biochemical verification:** none **Other relevant outcomes reported:** none	
Notes	**Notes:** quitting outcomes were collected but not reported **Funding source:** not reported **Author conflicts of interest:** not reported	
***Risk of bias***		
**Bias**	**Authors' judgement**	**Support for judgement**
Random sequence generation (selection bias)	Low risk	Quote: "Group assignment was done using an online random number generator"
Allocation concealment (selection bias)	Unclear risk	Quote: "Group assignment was done using an online random number generator" However, it is unclear if this was sufficient to conceal allocation.
Blinding of outcome assessment (detection bias) All outcomes	High risk	Biochemical validation not used and unequal levels of contact between study arms
Incomplete outcome data (attrition bias) All outcomes	Unclear risk	Attrition at 12‐month follow‐up not reported
Selective reporting (reporting bias)	Unclear risk	No protocol available
Other bias	High risk	Waitlist control ‐ participants in the control arm may have delayed quitting, knowing that they would be receiving an intervention at a later date. This has the potential to inflate the reported effect of the intervention.

**Table:** Singh 2014

***Study characteristics***		
Methods	**Study design:** RCT **Location:** not reported **Setting:** community **Recruitment:** by referrals from families, supported living and group home supervisors, and primary care physicians **Study dates:** not reported	
Participants	N = 51 **Specialist population?:** adults with mild intellectual disability **Definition of smoker used:** unclear **Participant characteristics:** 18% female; average age: 34 years; average cpd: 12; average years smoking: 16	
Interventions	**Comparator: **treatment as usual, including motivational therapies, behaviour therapies, NRTs, non‐nicotine medicines, or a combination of the above **Mode of delivery:** not reported **Intensity:** unclear, varied across participants **Pharmacotherapy:** none specifically provided, although for some usual care included NRT **Type of therapist/provider:** community therapists **BCTs:** 2.3 Self‐monitoring (behaviour), 11.1 Pharmacological support **Intervention: **training in the use of mindfulness and meditation techniques **Mode of delivery:** face‐to‐face (individual or small groups) or via Skype **Intensity:** 2 training sessions (1 x 1 h, 1 x 45 min), 10 supervised practice sessions (x 30 min over 5 days), weekly contact with trainer **Pharmacotherapy:** none **Type of therapist/provider:** trainer with a 35‐year history of service provision to people with intellectual and developmental disabilities and long‐standing personal meditation practice, clinical expertise, and experience in mindful service delivery to individuals at all levels of intellectual functioning **BCTs:** 1.9 Commitment, 2.3 Self‐monitoring of behaviour, 12.4 Distraction, 12.6 Body changes	
Outcomes	**Definition of abstinence:** 7‐day point prevalence **Longest follow‐up:** 12 months **Biochemical verification:** none **Other relevant outcomes reported:** none	
Notes	**Relevant comparisons:** mindfulness and meditation training vs treatment as usual **Funding source:** not reported **Author conflicts of interest:** not reported	
***Risk of bias***		
**Bias**	**Authors' judgement**	**Support for judgement**
Random sequence generation (selection bias)	High risk	Randomisation was via alternate placement in the experimental and control groups
Allocation concealment (selection bias)	Unclear risk	Concealment not reported
Blinding of outcome assessment (detection bias) All outcomes	High risk	Abstinence self‐reported, differing levels of face‐to‐face contact between arms
Incomplete outcome data (attrition bias) All outcomes	Unclear risk	Only reported attrition during treatment
Selective reporting (reporting bias)	Unclear risk	No protocol available

**Table:** Vidrine 2016

***Study characteristics***		
Methods	**Study design:** RCT **Location:** USA **Setting:** community **Recruitment:** local print media **Study dates:** 2007‐10	
Participants	N = 412 **Specialist population?:** no **Definition of smoker used:** average of ≥ 5 cpd for the past year, expired air CO ≥ 8 ppm **Participant characteristics:** 55% female; average age: 49 years; 42% white; 9% less than high school education; 58% household income < USD 30,000/year; average cpd: 20; nicotine dependence: 39% first cigarette within 5 min of waking; 17% history of depression	
Interventions	**Comparator 1: **usual care (intended to be equivalent to the intervention a smoker might receive when asking a healthcare provider for help) + self‐help materials **Mode of delivery:** face‐to‐face (individual), written materials **Intensity:** 4 sessions (x 5‐10 min) **Pharmacotherapy:** 6 weeks of nicotine patches, adjusted to individual cpd **Type of therapist/provider:** master's‐level therapists **BCTs:** 1.1 Goal setting (behaviour), 1.2 Problem solving, 3.1 Social support (unspecified), 4.1 Instruction on how to perform behaviour: self‐help, 11.1 Pharmacological support **Comparator 2: **CBT using a problem‐solving/coping skills training approach based on relapse prevention theory and national guidelines + self‐help materials **Mode of delivery:** face‐to‐face (group), written materials **Intensity:** 8 sessions (x 2 h) **Pharmacotherapy:** 6 weeks of nicotine patches, adjusted to individual cpd **Type of therapist/provider:** master's‐level therapists **BCTs:** 1.1 Goal setting (behaviour), 1.2 Problem solving, 4.1 Instruction on how to perform behaviour: self‐help, 5.1 Information about health consequences, 9.2 Pros and cons, 11.1 Pharmacological support **Intervention: **mindfulness‐based addiction treatment (MBAT), which integrates mindfulness‐based stress reduction (MBSR) with a cognitive behavioural/relapse prevention theory‐based approach + self‐help materials **Mode of delivery:** face‐to‐face (group), written materials **Intensity:** 8 sessions (x 2 h) **Pharmacotherapy:** 6 weeks of nicotine patches, adjusted to individual cpd **Type of therapist/provider:** master's‐level therapists skilled in delivering MBSR **BCTs:** 1.1 Goal setting (behaviour), 1.2 Problem solving, 3.1 Social support (unspecified), 4.1 Instruction on how to perform behaviour: self‐help, 11.1 Pharmacological support, 11.2 Reduce negative emotions, 12.6 Body changes	
Outcomes	**Definition of abstinence:** 7‐day point prevalence **Longest follow‐up:** 6 months post‐quit day **Biochemical verification:** CO < 6 ppm. or salivary cotinine < 20 ng/mL if did not attend follow‐up in person **Other relevant outcomes reported:** depression, stress, positive and negative affect	
Notes	**Relevant comparisons:** mindfulness‐based addiction treatment + self‐help materials + NRT vs CBT + self‐help materials + NRT mindfulness‐based addiction treatment + self‐help materials + NRT vs usual care + self‐help materials + NRT **Funding source:** National Institute on Drug Abuse, Centers for Disease Control and Prevention, National Cancer Institute, National Center for Complementary and Integrative Health, and Oklahoma Tobacco Settlement Endowment Trust **Author conflicts of interest:** not reported	
***Risk of bias***		
**Bias**	**Authors' judgement**	**Support for judgement**
Random sequence generation (selection bias)	Unclear risk	Method not reported
Allocation concealment (selection bias)	Unclear risk	Concealment not reported
Blinding of outcome assessment (detection bias) All outcomes	Low risk	Abstinence biochemically verified
Incomplete outcome data (attrition bias) All outcomes	Low risk	At 6‐month follow‐up 33.1% (51/154) were lost to follow‐up in the mindfulness‐based addiction treatment group, 34.8% (54/155) in the CBT group and 35.9% (37/103) in the usual care group
Selective reporting (reporting bias)	Low risk	Prespecified outcomes reported

**Table:** Weng 2021

***Study characteristics***		
Methods	**Study design:** RCT **Location:** Hong Kong **Setting:** workplace **Recruitment:** from companies in “women‐related industries such as … beauty and retail sectors” or individually through outreaching recruitment sessions **Study dates:** 2015‐16	
Participants	N = 213 **Specialist population?:** women **Definition of smoker used:** ≥ 1 cpd in the past 3 months **Participant characteristics:** 100% female; average age: 34 years; 90% secondary education or below; 72% monthly income ≤ HKD 20,000; average cpd: 11; nicotine dependence: 19% FTND ≥ 6	
Interventions	All participants were given a 50‐page self‐help smoking cessation booklet (about 9000 words) tailored to women smokers in workplaces and offered an optional 1‐h health talk (on the hazards of smoking and benefits and methods of quitting) **Comparator: **brief advice to follow the booklet’s advice **Mode of delivery:** face‐to‐face (individual), written materials **Intensity:** 1 session (presumably brief) + optional 1‐h health talk **Pharmacotherapy:** none **Type of therapist/provider:** trained counsellors **BCTs:** 4.1 Instruction on how to perform the behaviour, 5.1 Information about health consequences **Intervention: **brief mindfulness training **Mode of delivery:** face‐to‐face (group), written materials **Intensity:** 2 sessions (x 2 h) over 2 weeks + optional 1‐h health talk **Pharmacotherapy:** none **Type of therapist/provider:** certified clinical therapist **BCTs:** 4.1 Instruction on how to perform behaviour (self‐help guide), 5.1 Information about health consequences, 11.2 Reducing negative emotions, 12.6 Body changes	
Outcomes	**Definition of abstinence:** 7‐day point prevalence **Longest follow‐up:** 6 months **Biochemical verification:** CO < 4 ppm and salivary cotinine < 10 ng/mL **Other relevant outcomes reported:** none	
Notes	**Relevant comparisons:** brief mindfulness training + self‐help materials vs brief advice + self‐help materials **Funding source:** Lok Sin Tong Benevolent Society Kowloon **Author conflicts of interest:** “The authors declared that there is no conflict of interest.”	
***Risk of bias***		
**Bias**	**Authors' judgement**	**Support for judgement**
Random sequence generation (selection bias)	Unclear risk	Method not reported
Allocation concealment (selection bias)	Low risk	Quote: “The allocation sequence was generated by a researcher who was not involved in participant recruitment.”
Blinding of outcome assessment (detection bias) All outcomes	Low risk	Abstinence biochemically verified
Incomplete outcome data (attrition bias) All outcomes	Low risk	At 6‐month follow‐up 24.6% (28/114) were lost to follow‐up in the intervention group and 20.2% (20/99) in the control group
Selective reporting (reporting bias)	Low risk	Prespecified outcomes reported

**ACT:** acceptance and commitment therapy; **BCT:** behaviour change techniques; **CBT:** cognitive behavioural therapy; **cpd:** cigarettes per day; **FTND:** Fagerström Test for Nicotine Dependence (Heatherton 1991); **HSI:** Heaviness of Smoking Index (Heatherton 1989); **NRT:** nicotine replacement therapy; **ppm:** parts per million; **PTSD:** post‐traumatic stress disorder; **RCT:** randomised controlled trial; **SES:** socioeconomic status

### Characteristics of excluded studies [ordered by study ID]

**Table:**

Study	Reason for exclusion
Aggarwal 2017	Ineligible follow‐up period
Arcari 1997	Ineligible follow‐up period
Arora 2013	Not possible to isolate the mindfulness element
Baruffi 2014	Ineligible patient population
Bloom 2017	Ineligible study design
Bowen 2009	Ineligible follow‐up period
Brewer 2011	Ineligible follow‐up period
Bricker 2014b	Ineligible follow‐up period
Chirikos 2004	Ineligible patient population
Cropley 2007	Ineligible follow‐up period
Davis 2013	Ineligible follow‐up period
Davoudi 2017	Ineligible follow‐up period
Elibero 2011	Ineligible follow‐up period
Elwafi 2013	Ineligible follow‐up period
Gifford 2011	Not possible to isolate the mindfulness element
Heffner 2013	Ineligible follow‐up period
Hemenway 2021	Ineligible follow‐up period
Hernandez‐Lopez 2009	Ineligible study design
Janes 2019	Ineligible follow‐up period
Jang 2019	Ineligible intervention
Jones 2015	Ineligible follow‐up period
Jones 2017	Not possible to isolate the mindfulness element
Karelka 2020	Ineligible follow‐up period
Kochupillai 2005	Ineligible study design
Luberto 2016	Ineligible follow‐up period
Luk 2019	Not possible to isolate the mindfulness element
Luo 2018	Ineligible follow‐up period
Minami 2018	Ineligible follow‐up period
Mineyama 2019	Ineligible study design
Mujcic 2018	Not possible to isolate the mindfulness element
NCT01314378	Ineligible follow‐up period
NCT04038255	Ineligible follow‐up period
Otto 2020	Ineligible follow‐up period
Pakhale 2014	Ineligible study design
Rogojanski 2011a	Ineligible follow‐up period
Rogojanski 2011b	Ineligible follow‐up period
Ruscio 2016	Ineligible follow‐up period
Sarkar 2017	Not possible to isolate the mindfulness element
Schuman‐Olivier 2014	Ineligible follow‐up period
Shahab 2013	Ineligible follow‐up period
Sharma 2013	Ineligible study design
Sidhu 2016	Ineligible study design
Spears 2019	Ineligible follow‐up period
Sussman 2004	Not possible to isolate the mindfulness element
Tang 2013	Ineligible outcomes
Ussher 2009	Ineligible follow‐up period
Zeng 2016	Ineligible study design

### Characteristics of studies awaiting classification [ordered by study ID]

**Table:** Pumariega 2020

Methods	**Study design:** RCT **Location:** Brazil **Setting:** Psychosocial Care Center for Alcohol and Drugs **Recruitment:** from smokers seeking treatment at a substance centre **Study dates:** 2017
Participants	N = 52 **Specialist population?:** smokers seeking treatment **Definition of smoker used:** unclear **Participant characteristics:** 83% female; average age: 49 years; 74% white; 69% lower middle socioeconomic class; 43% high nicotine dependence
Interventions	**Comparator:** usual care based on CBT **Intervention:** mindfulness‐based relapse prevention
Outcomes	**Definition of abstinence:** unclear **Longest follow‐up:** unclear **Biochemical verification:** CO **Other relevant outcomes reported:** depression, anxiety
Notes	Follow‐up period unclear

**CBT:** cognitive behavioural therapy; **RCT:** randomised controlled trial

### Characteristics of ongoing studies [ordered by study ID]

**Table:** CTRI/2020/01/022692

Study name	Yoga as a complementary intervention for tobacco cessation: a PROBE trial
Methods	**Study design:** RCT **Location:** India **Setting:** Centre of Integrative Medicine and Research, All India Institute of Medical Sciences **Recruitment:** not reported **Study dates:** 2020‐23 (estimated)
Participants	Target N = 200 **Specialist population?:** no **Definition of smoker used:** ≥ 5 cpd for at least the past year
Interventions	**Comparator:** exercise + wellness programme **Mode of delivery:** face‐to‐face, video, audio recordings and written materials **Intensity:** 4 supervised sessions in the first week followed by 1 weekly session for 7 weeks **Pharmacotherapy:** **Type of therapist/provider:** institutionally certified yoga therapist + study wellness counsellor or other healthcare professional (e.g. psychologist) **Intervention:** yoga + wellness programme **Mode of delivery:** face‐to‐face, video and telephone **Intensity:** 4 supervised sessions in the first week followed by weekly sessions for 7 weeks **Pharmacotherapy:** none **Type of therapist/provider:** institutionally certified yoga therapist + study wellness counsellor or other healthcare professional (e.g. psychologist) **BCTs:** 4.1 Instruction on how to perform behaviour, 5.1 Information about health consequences, 8.6 Generalisation of a target behaviour, 12.6 Body changes
Outcomes	**Definition of abstinence:** unclear **Longest follow‐up:** 6 months **Biochemical verification:** salivary cotinine and CO (threshold not reported) Other relevant outcomes: depression, anxiety, quality of life
Starting date	2020
Contact information	Gautam Sharma, All India Institute of Medical Sciences (AIIMS)
Notes	**Contacted PI for update:** no update to report **Funding source:** Centre for Integrative Medicine and Research (CIMR) Institute Name: All India Institute of Medical Sciences (AIIMS), New Delhi **Author conflicts of interest:** not reported

**Table:** NCT01098955

Study name	Smoking cessation treatment for head & neck cancer patients
Methods	**Study design:** RCT **Location:** USA **Setting:** cancer centre **Recruitment:** from patients planning to or currently undergoing treatment, or in follow‐up care **Study dates:** 2010‐2021 (estimated)
Participants	**Specialist population?:** current or history of head and neck, lung, breast, gastrointestinal, or genitourinary cancer **Definition of smoker used:** ≥ 1 cpd
Interventions	**Comparator:** motivational and behavioural counselling **Mode of delivery:** not reported **Intensity:** 6 counselling sessions (x 1 h) delivered over a 5‐week period **Pharmacotherapy:** varenicline (2 mg daily for 12 weeks) **Type of therapist/provider:** not reported **BCTs:** 3.1 Social support (unspecified), 11.1 Pharmacological support **Intervention:** ACT **Mode of delivery:** not reported **Intensity:** 6 counselling sessions (x 1 h) delivered over a 5‐week period **Pharmacotherapy:** varenicline (2 mg daily for 12 weeks) **Type of therapist/provider:** not reported **BCTs:** 1.9 Commitment, 3.1 Social support (unspecified): counselling, 11.1 Pharmacological support
Outcomes	**Definition of abstinence:** unclear **Longest follow‐up:** 26 weeks after target quit date **Biochemical verification:** unclear **Other relevant outcomes:** none
Starting date	2010
Contact information	Jan Blalock, PhD M.D. Anderson Cancer Center
Notes	**Contacted PI for update:** no update to report **Funding source:** not reported **Author conflicts of interest:** not reported

**Table:** NCT01982110

Study name	A mindfulness based application for smoking cessation (MBSC)
Methods	**Study design:** RCT **Location:** USA **Setting:** community **Recruitment:** not reported **Study dates:** 2013‐18
Participants	N = 5 **Specialist population?:** no **Definition of smoker used:** ≥ 5 cpd
Interventions	**Comparator:** behavioural smoking cessation (NCI Quit Pal app) **Mode of delivery:** smartphone app **Intensity:** not reported **Pharmacotherapy:** none **Type of therapist/provider:** n/a **BCTs:** unclear **Intervention:** mindfulness‐based therapy (Craving to Quit app) **Mode of delivery:** smartphone app **Intensity:** not reported **Pharmacotherapy:** none **Type of therapist/provider:** n/a **BCTs:** 12.6 Body changes
Outcomes	**Definition of abstinence:** unclear **Longest follow‐up:** 6 months **Biochemical verification:** CO (threshold not reported) **Other relevant outcomes:** none
Starting date	2013
Contact information	Jennifer K Penberthy, PhD University of Virginia
Notes	**Contacted PI for update:** no response **Funding source:** not reported **Author conflicts of interest:** not reported

**Table:** NCT02037360

Study name	Mobile mindfulness training for smoking cessation
Methods	**Study design:** RCT **Location:** USA **Setting:** not reported **Recruitment:** not reported **Study dates:** 2015‐17
Participants	N = 1251 **Specialist population?:** no **Definition of smoker used:** ≥ 5 cpd with < 3 months' abstinence in the past year
Interventions	**Comparator:** standard smartphone app to support smokers to quit **Mode of delivery:** smartphone app **Intensity:** not reported **Pharmacotherapy:** none **Type of therapist/provider:** n/a **BCTs:** 1.1 Goal setting (behaviour), 1.3 Goal setting (outcome), 2.3 Self‐monitoring (behaviour), 3.1 Social support (unspecified) **Intervention:** smartphone app that provides training in mindfulness for smoking cessation **Mode of delivery:** smartphone app **Intensity:** 22 modules (x 10‐15 minutes each) + 5 bonus modules available upon completion of earlier modules **Pharmacotherapy:** none **Type of therapist/provider:** n/a **BCTs:** 1.1 Goal setting (behaviour), 1.2 Problem solving, 1.3 Goal setting (outcome), 2.3 Self‐monitoring (behaviour), 12.6 Body changes
Outcomes	**Definition of abstinence:** 7‐day point prevalence **Longest follow‐up:** 6 months **Biochemical verification:** not reported **Other relevant outcomes:** none
Starting date	2015
Contact information	Judson Brewer, MD PhD University of Massachusetts, Worcester
Notes	**Contacted PI for update:** data were never analysed **Funding source:** not reported **Author conflicts of interest:** not reported

**Table:** NCT02421991

Study name	Telephone‐delivered interventions for smoking cessation (TALK)
Methods	**Study design:** RCT **Location:** USA **Setting:** not reported **Recruitment:** not reported **Study dates:** 2015‐19
Participants	N = 1275 **Specialist population?:** no **Definition of smoker used:** ≥ 10 cpd for at least the past 12 months
Interventions	**Comparator:** CBT **Mode of delivery:** telephone **Intensity:** 5 weekly sessions **Pharmacotherapy:** NRT **Type of therapist/provider:** not reported **BCTs:** 3.1 Social support **Intervention:** Acceptance and Commitment Therapy (ACT) **Mode of delivery:** telephone **Intensity:** 5 weekly sessions **Pharmacotherapy:** NRT **Type of therapist/provider:** not reported **BCTs:** 3.1 Social support (note: full details of therapy not provided)
Outcomes	**Definition of abstinence:** 30‐day point prevalence **Longest follow‐up:** 12 months **Biochemical verification:** not reported Other relevant outcomes: none
Starting date	2015
Contact information	Jonathan B Bricker, Ph.D. Fred Hutchinson Cancer Research Center
Notes	**Contacted PI for update:** no update to report **Funding source:** not reported **Author conflicts of interest:** not reported

**Table:** NCT03253445

Study name	Individual acceptance and commitment therapy (ACT) for smoking cessation for schizophrenic patients
Methods	**Study design:** RCT **Location:** Hong Kong **Setting:** not reported **Recruitment:** not reported **Study dates:** 2014‐18 (estimated)
Participants	N = 160 (target) **Specialist population?:** diagnosed schizophrenia **Definition of smoker used:** ≥ 1 cpd
Interventions	All participants are given a brief educational talk on encouraging quitting smoking (about 5 min) and a self‐help leaflet on smoking cessation **Comparator:** ACT + brief advice + self‐help materials **Mode of delivery:** face‐to‐face, written materials **Intensity:** 10 sessions (x 20‐30 min) **Pharmacotherapy:** none **Type of therapist/provider:** n/a **BCTs:** 1.9 Commitment, 4.1 Instruction on how to perform the behaviour **Intervention:** social support + brief advice + self‐help materials **Mode of delivery:** face‐to‐face, written materials **Intensity:** 10 sessions (x 5 min) **Pharmacotherapy:** none **Type of therapist/provider:** n/a **BCTs:** 3.1 Social support (unspecified), 4.1 Instruction on how to perform the behaviour
Outcomes	**Definition of abstinence:** 7‐day point prevalence **Longest follow‐up:** 6 months **Biochemical verification:** CO (threshold not reported), urinary cotinine (threshold not reported) **Other relevant outcomes:** none
Starting date	2014
Contact information	Dr. Yim Wah Mak, Assistant Professor, The Hong Kong Polytechnic University
Notes	**Contacted PI for update:** no response **Funding source:** not reported **Author conflicts of interest:** not reported

**ACT:** acceptance and commitment therapy; **BCT:** behaviour change techniques; **CBT:** cognitive behavioural therapy; **cpd:** cigarettes per day; **n/a:** not applicable; **NRT:** nicotine replacement therapy; **PI:** principal investigator; **RCT:** randomised controlled trial

## Differences between protocol and review

- We specified in our protocol that we would include measures of depression, anxiety, quality of life, and stress as indicators of mental health and well‐being. We also included data on positive and negative affect as several of our included studies had reported on these outcomes.
- We originally proposed that in the case of cluster‐RCTs, we would extract a direct estimate of the required effect from an analysis that properly accounted for the cluster design, and where such data were unavailable, we would perform an approximately correct analysis if we could extract the intracluster correlation coefficient (ICC). However, the one cluster‐RCT we identified as suitable for inclusion did not present an analysis adjusting for the clustering effect or report an ICC. Therefore, we used unadjusted data for the primary analysis and performed a sensitivity analysis where we estimated the ICC (0.03), based on the ICC reported in other smoking cessation studies (Fanshawe 2017), and adjusted the analysis on this basis.
- We originally planned to conduct subgroup analyses categorising studies by (broad) intervention type, the type/intensity of control treatment received, population type, baseline motivation to quit, baseline mental health, number of intervention behaviour change techniques, mode of intervention delivery, and type of therapist. Given the studies covered a diverse set of interventions, we did not consider it appropriate to pool all studies in a single meta‐analysis, so we stratified our pooled analyses by intervention type. It was then possible to conduct subgroup analyses by the type and intensity of control treatment received and mode of intervention delivery. 
